# Hetero‐Diatomic CoN_4_‐NiN_4_ Site Pairs with Long‐Range Coupling as Efficient Bifunctional Catalyst for Rechargeable Zn–Air Batteries

**DOI:** 10.1002/advs.202310231

**Published:** 2024-03-30

**Authors:** Yue Yang, Bin Li, Yining Liang, Wenpeng Ni, Xuan Li, Gengzhe Shen, Lin Xu, Zhengjian Chen, Chun Zhu, Jin‐Xia Liang, Shiguo Zhang

**Affiliations:** ^1^ Zhuhai Institute of Advanced Technology, Shenzhen Institutes of Advanced Technology Chinese Academy of Sciences Zhuhai 519000 China; ^2^ School of Chemistry and Chemical Engineering Guizhou University Guiyang 550025 China; ^3^ College of Materials Science and Engineering, State Key Laboratory of Advanced Design and Manufacturing for Vehicle Body Hunan University Changsha 410004 China

**Keywords:** density functional theory, diatomic catalyst, oxygen evolution reaction, oxygen reduction reaction, rechargeable Zn–air batteries

## Abstract

In this study, Co/Ni‐NC catalyst with hetero‐diatomic Co/Ni active sites dispersed on nitrogen‐doped carbon matrix is synthesized via the controlled pyrolysis of ZIF‐8 containing Co^2+^ and Ni^2+^ compounds. Experimental characterizations and theoretical calculations reveal that Co and Ni are atomically and uniformly dispersed in pairs of CoN_4_‐NiN_4_ with an intersite distance ≈0.41 nm, and there is long‐range *d*–*d* coupling between Co and Ni with more electron delocalization for higher bifunctional activity. Besides, the in situ grown carbon nanotubes at the edges of the catalyst particles allow high electronic conductivity for electrocatalysis process. Electrochemical evaluations demonstrate the superior ORR and OER bifunctionality of Co/Ni‐NC catalyst with a narrow potential gap of only 0.691 V and long‐term durability, significantly prevailing over the single‐atom Co‐NC and Ni‐NC catalysts and the benchmark Pt/C and RuO_2_ catalysts. Co/Ni‐NC catalyzed Zn–air batteries achieve a high specific capacity of 771 mAh g^−1^ and a long continuous operation period up to 340 h with a small voltage gap of ≈0.65 V, also much superior to Pt/C‐RuO_2_.

## Graphical abstract

CoN_4_‐NiN_4_ site pairs with long‐range coupling are atomically dispersed on nitrogen‐doped carbon matrix, and the resultant catalyst is found to show excellent ORR/OER bi‐functionality and used as cathode catalyst for high‐performance rechargeable Zn–air battery.

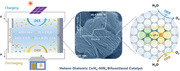

## 1 Introduction

Rechargeable Zn–air batteries are widely recognized as one of the most promising next‐generation electrochemical energy conversion and storage systems, due to their high energy capacity (820 mAh g^−1^), low cost, and safety benefits.^[^ ^1^ ^]^ Nevertheless, the sluggish kinetics of the air cathodes for both oxygen reduction reaction (ORR) and oxygen evolution reaction (OER) has severely impeded their practical application.^[^ ^2^ ^]^ Generally, ORR in aqueous alkaline electrolytes follows either a two‐electron process yielding superoxide (O_2_^−^) or a four‐electron process producing hydroxide (OH^−^), and the latter is preferable due to its higher efficiency and non‐corrosive property.^[^ ^3^ ^]^ The alkaline OER mechanism involves the formation of metal oxyhydroxides (M‐OOH), which finally decompose into O_2_ and H_2_O.^[^ ^4^ ^]^ Both ORR and OER are heterogeneous electrochemical processes that take place at the tri‐phase boundary (solid catalyst, gas oxygen, and liquid electrolyte), resulting in high polarization resistance and low oxygen reactivity,^[^ ^5^ ^]^ which need to be overcome by electrocatalysts.^[^ ^6^ ^]^ Although the precious metal‐based catalysts Pt/C and RuO_2_/IrO_2_ have demonstrated superior performance for ORR and OER respectively, they still suffer from high cost, resource scarcity, poor stability, and inferior bifunctionality.^[^ ^7^ ^]^ Therefore, the research and development of advanced bifunctional electrocatalysts with high performance and low cost have become a research hotspot for Zn–air batteries.^[^ ^8^ ^]^

Single‐atom catalysts (SACs) have recently demonstrated superior performance in various catalytic reactions, due to the highly dispersed active sites, maximum atomic utilization, unique geometric configuration, and excellent activity and selectivity.^[^ ^9^ ^]^ However, the atomically dispersed metal atoms with extremely high specific surface energy tend to migrate and agglomerate. Thus, fabricating SACs with dense dispersion remains challenging.^[^ ^10^ ^]^ Heteroatom‐doped carbon matrix containing intrinsic defect traps and coordinating heteroatoms can efficiently limit the undesired migration, aggregation and corrosion of the atomically dispersed metal atoms.^[^ ^11^ ^]^ In addition, the doping of heteroatoms (such as N, P, and B) may alter the local electronic structure of carbon nanocones, expose more active sites, and thus greatly improve the catalytic performance of SACs.^[^ ^11^, ^12^ ^]^ Among the reported oxygen reaction catalysts, transition metal‐nitrogen‐doped carbon (M‐N‐C, M: Fe, Co, and Ni, etc.) materials are very competitive.^[^ ^13^ ^]^ For example, Fe‐N‐C catalysts have excellent ORR activity, due to the super active FeN_4_ sites.^[^ ^14^ ^]^ However, Fe‐N‐C catalysts are subjected to poor durability associated with Fe demetallation via the Fenton reaction,^[^ ^15^ ^]^ and relatively inferior OER activity.^[^ ^16^ ^]^ Meantime, the single Co and Ni active sites are only effective for ORR and OER, respectively, with inferior bifunctionality.^[^ ^17^ ^]^

As a further extension of SACs, diatomic catalysts (DACs) essentially inherit the exceptional merits of SACs, and the binary combination of atomic active sites enables superior structural and compositional flexibility, synergistic action, and new catalytic mechanism.^[^ ^18^ ^]^ The combination pattern (such as distance) between hetero‐bimetallic atoms is critical for the catalytic activity of DACs.^[^ ^18^, ^19^ ^]^ The corresponding distances usually range from 2 to 4 Å, and there exists a local interaction structure between the diatomic sites.^[^ ^19b^ ^]^ For example, the adjacent Fe‐Ni dual sites embedded on nitrogen‐doped carbon hollow spheres showed excellent bifunctionality, and Ni and Fe are the active sites for OER and ORR, respectively.^[^ ^20^ ^]^ Theoretical calculations suggested that the diatomic pairs (such as Fe‐Co or Zn‐Co) can significantly elongate the O─O bond and thus facilitate the bond cleavage during the oxygen activation.^[^ ^21^ ^]^ Meanwhile, the neighboring Co‐Ni dual sites were supposed to have synergistic effect for optimizing the adsorption/desorption features and decreasing the overall reaction barriers for the reversible oxygen redox reactions.^[^ ^22^ ^]^ When spatially separated by one or two organic atoms, there are also long‐range synergistic interactions between the hetero‐diatomic metal sites.^[^ ^23^ ^]^ For example, the Cu and Zn atoms with 4.1 Å distance and non‐covalent interaction anchored on nitrogen‐doped carbon can enhance the orbital hybridization between metallic 3d and O 2p orbitals of the adsorbent oxygen, and therefore activate O─O bond.^[^ ^23b^ ^]^ When the distance increases further, DACs should be regarded as two individual SACs, but the DACs usually reveal non‐linear combination performance of the two SACs.^[^ ^19^, ^24^ ^]^ So far, the chemical interactions and synergistic effects of DACs remain controversial and ambiguous,^[^ ^25^ ^]^ and the precisely‐controlled synthesis of well‐defined DACs with hetero‐bimetallic active sites for high bifunctional performance is also a great challenge.^[^ ^26^ ^]^

Herein, Co/Ni‐NC catalyst with hetero‐diatomic CoN_4_‐NiN_4_ site pairs atomically dispersed on nitrogen‐doped carbon matrix was controllably synthesized and found to have superior ORR and OER bifunctionality. Thus Co/Ni‐NC was used as catalyst for assembling high‐performance Zn–air batteries, better than Pt/C‐RuO_2_‐based batteries. Theoretical calculations were carried out to understand the synergistic mechanism of the hetero‐diatomic site pairs on improving both the ORR and OER activities.

## 2 Results and Discussion

### 2.1 Catalysts Structure and Characterization

**Figure 1:**
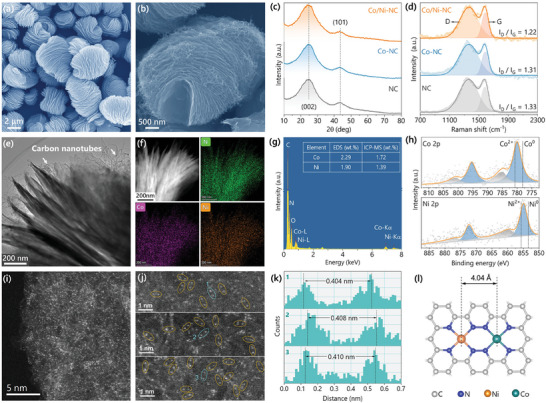
SEM images of a) CoNi‐ZIF‐8 and b) its pyrolysis product Co/Ni‐NC. c) XRD and d) Raman spectra of Co/Ni‐NC, Co/NC, and NC. e) STEM images and f) EDS mappings for N, Co, and Ni elements of Co/Ni‐NC. g) EDS spectrum of Co/Ni‐NC, where the inset table contains the Ni and Co contents obtained by EDS and ICP‐MS methods. h) High‐resolution XPS of the Co 2p and Ni 2p for the Co/Ni‐NC. i,j) Aberration‐corrected HAADF‐STEM images of Co/Ni‐NC. k) The corresponding diatomic distances marked in (j). l) Schematic model of neighboring CoN_4_ and NiN_4_ sites optimized by DFT calculations (the orange, green, blue, and gray spheres represent Ni, Co, N, and C atoms, respectively).

Co/Ni‐NC catalyst was facilely synthesized through the direct pyrolysis of ZIF‐8 containing Co and Ni species without any further treatment (see detail in Supporting Information). Co‐NC, Ni‐NC, and NC (metal‐free nitrogen‐doped carbon) were also synthesized for comparison. Field‐emission scanning electron microscopy (FE‐SEM, **Figure** **1**a; Figure S1a,b, Supporting Information) illustrates the layer‐by‐layer stacking structure of ZIF‐8 with an average layer diameter of ≈4 µm and an average layer thickness of ≈30 nm. The laminated structure remains intact after combining Co and Ni species, as confirmed by X‐ray diffraction analysis (XRD, Figure S1c,d, Supporting Information). ZIF‐8 serves as a self‐sacrificial template during the pyrolysis, and the obtained catalysts almost perfectly inherit the layered structure, as shown in SEM (Figure 1b) and transmission electron microscopy (TEM, Figure S2, Supporting Information). According to high‐resolution TEM (HRTEM, Figure S2c,d, Supporting Information), the carbon materials are comprised of mostly amorphous and partially graphitized carbon with a typical (002) interplanar spacing of 0.34 nm. HRTEM results also reveal the presence of nanopores, which are induced by the evaporation of Zn species in the process of pyrolysis,^[^ ^27^ ^]^ and the absence of metal nanoclusters or nanoparticles indicates their very high dispersion. The specific surface area of Co/Ni‐CN was measured to be 734 m^2^ g^−1^, which is close to the recent literature results, such as SiO_2_@Fe‐ZIF‐8/67 derived A‐FeCo@NCNs, 809 m^2^ g^−1^),^[^ ^1d^ ^]^ ES‐Co/Zn‐CN_ZIF_ (596 m^2^ g^−1^),^[^ ^8d^ ^]^ and ZIF‐8 derived Fe/Zn‐CHNC, FeCo‐N‐C, Fe‐Nx/C (1351, 428, and 662 m^2^ g^−1^, respectively).^[^ ^28^ ^]^ According to the N_2_ adsorption–desorption isotherms and pore size distribution shown in Figure S3 (Supporting Information), the high surface area of Co/Ni‐CN is mainly derived from micropores in the range of 0.4–0.6 nm (insert in Figure S3b, Supporting Information), agreeing well with the observation in HRTEM images. Figure 1c presents the XRD patterns, where only two broad peaks located at 25° and 44°, corresponding to the (002) and (101) carbon diffractions, are detected for all the measured materials. This result also reflects the partial carbon graphitization and the absence of metal crystalline. In the Raman spectra (Figure 1d), typical D (disorder) and G (graphite) bands were found at 1350 and 1580 cm^−1^, respectively. Accordingly, the tail of the D band extends to the center of the G band, and the D band peak is unaffected by overlapping. Therefore, the fitted G band height is lower than the original value, and the fitted D peak is as high as the original value.^[^ ^29^ ^]^ The intensity ratio (I_D_/I_G_) generally declines in the order of NC (1.33) > Co‐NC (1.31) > Co/Ni‐NC (1.22), probably because the metals may serve as catalysts to promote the transformation of amorphous carbon to graphitic structure.^[^ ^30^ ^]^ This result could be also verified by the observation of carbon nanotubes (∼20 nm in diameter) at the edges, as shown in Figure 1e and Figure S2a,b (Supporting Information), which should form and grow at the metal active sites.^[^ ^30e^ ^]^ The coexistence of carbon nanotubes and graphitized carbon allows high electron conductivity for good electrocatalytic activity. Energy dispersive X‐ray spectroscopy (EDS, Figure 1f) images show the homogenous distribution of N, Ni and Co elements throughout the entire carbon framework. According to the peak intensities of Co‐Kα at 6.93 keV and Ni‐Kα at 7.47 keV (Figure 1g), the Co and Ni loadings in the surface (few µm) of Co/Ni‐NC were estimated to be as high as 2.29 and 1.90 wt.%, respectively. The bulk contents of Co and Ni were determined to be 1.72 and 1.39 wt.% respectively, by an inductively coupled plasma mass spectrometry (ICP‐MS, see the table inset in Figure 1g). The difference between the surface loading and bulk content implies that Co and Ni elements in Co/Ni‐NC tend to appear in the surface rather than the bulk, which is beneficial for high catalytic activity.

The electronic states of the supported metals were measured by X‐ray photoelectron spectroscopy (XPS, Figure 1h). The binding energy of Co 2p_3/2_ in Co/Ni‐NC located between metallic Co^0^ (778.2 eV) and Co^2+^ (780.7 eV) indicates that the Co atoms are unsaturated and positively charged, in the form of Co^δ+^ (0 < δ < 2). Similarly, the Ni sites also exhibit an unsaturated valence state between Ni^0^ (853.5 eV) and Ni^2+^ (855.8 eV) in Co/Ni‐NC. The atomic level dispersion of Co and Ni metals over the N‐doped carbon matrix can be visually observed using aberration‐corrected atomic‐resolution HAADF‐STEM (Figures 1i,j), where the bright spots represent the transition metal atoms (Co and Ni) with higher electron densities than C and N atoms. The dense and uniform dispersion of Co/Ni atoms shown in Figures 1i,j agrees with the above high total metal loading (>3 wt.%). Additionally, the supported single metal atoms mainly exist in form of dual atom pairs with an average interatomic distance of 0.41 nm, as shown in Figure 1k. For more confirmation, different hetero‐diatomic configurations were theoretically simulated, as presented in Figure 1l and Figure S4 (Supporting Information). Among the optimized structures, the CoN_4_‐NiN_4_ configuration exhibits the most consistent intersite distance (4.04 Å) and metal coordination environment to the experimental results, namely the interatomic distance of 0.41 nm and coordination number of ≈4 (see X‐ray absorption edge spectrometric analysis below). Meantime, the adsorption energy (*E*_ads_) of Co/Ni simultaneously at the defects in N‐doped carbon was calculated to be much lower than that of single metal sites (Co/Ni: −14.58 eV, Co: −7.86 eV, Ni: −7.83 eV, Figure S5, Supporting Information), indicating the favorable formation of the CoN_4_‐NiN_4_ configuration.

**Figure 2:**
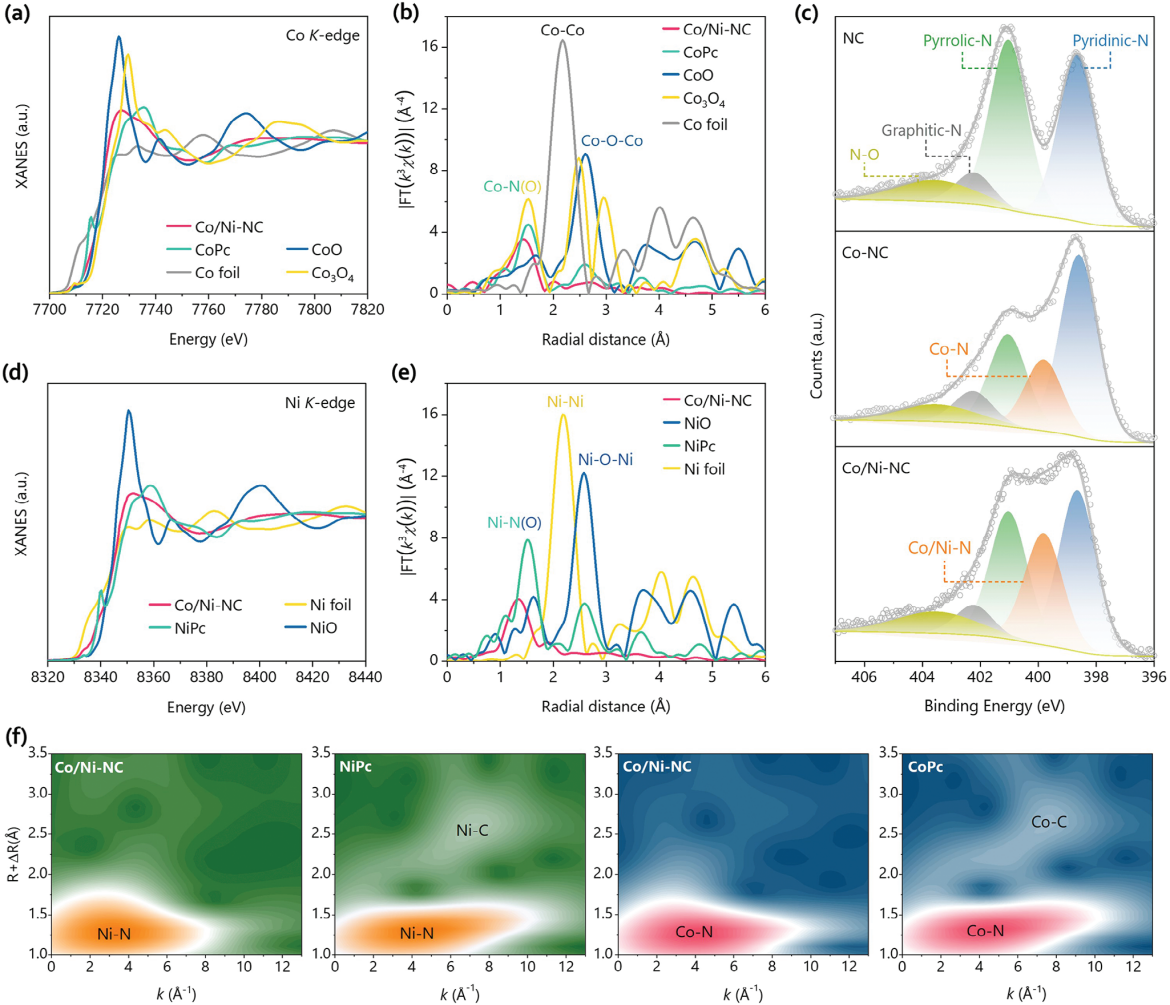
a) Normalized XANES spectra of Co/Ni‐NC, CoPc (cobalt phthalocyanine), CoO, Co_3_O_4_, and Co foil at Co K‐edge and b) Fourier‐transformation of the corresponding Co K‐edge EXAFS spectra at R space. c) N 1s XPS spectra of NC, Co‐NC, and Co/Ni‐NC. d) Normalized XANES spectra of Co/Ni‐NC, NiPc (nickel phthalocyanine), NiO, and Ni foil at Ni K‐edge and e) Fourier transformation of the corresponding Ni K‐edge EXAFS spectra. f) Wavelet‐transformation of the k^3^‐weighted Ni and Co K‐edge EXAFS signals of Co/Ni‐NC, NiPc, and CoPc.

To further identify the chemical state and coordination environment of Co and Ni atoms dispersed in the catalysts, X‐ray absorption near‐edge structure (XANES) and extended X‐ray absorption fine structure (EXAFS) spectrometry measurements were performed. As seen in **Figure** **2**a, the near‐edge absorption threshold of Co K‐edge in Co/Ni‐NC lies between those of Co foil (Co^0^) and CoO (Co^2+^), similar to that of CoPc (Co^+^). This result can be mutually corroborated with the above XPS analysis, namely Co^δ+^ (0 < δ < 2). Meantime, in the Co K‐edge EXAFS spectra shown in Figure 2b, the main peak of Co/Ni‐NC locates at 1.45 Å (without phase shift correction) in R space, close to the Co‐N peak of CoPc, and additionally, no significant Co─Co bond peak at 2.18 Å was observed, indicating that the Co species in Co/Ni‐NC mainly exists in the form of Co‐N coordination. The first shell EXAFS signal of Co in Co/Ni‐NC can be accurately fitted by the Co‐N path with the coordination number of 4.3 and the bond distance of 1.90 Å (Figure S6a and Table S1, Supporting Information). Thus one can see that the Co species is mainly anchored on the N‐doped carbon matrix in the CoN_4_ configuration. This result can be also witnessed by N 1s XPS spectra (Figure 2c), which were deconvolved into several peaks, including pyridinic N (398.6 eV), metal‐coordinated N (399.7 eV) and pyrrolic N (400.9 eV).^[^ ^31^ ^]^ The metal‐coordinated N 1s peak, which corresponds to the metal‐N coordination bond, is significant in Co‐NC and Co/Ni‐NC (especially for the latter) but absent in NC. Likewise, the Ni K‐edge XANES spectra shown in Figure 2d reveal that both the edge position and the peak intensity of Co/Ni‐NC locate between those of Ni foil and NiO, indicating that the valence of the Ni atoms in Co/Ni‐NC is between Ni^0^ and Ni^2+^, in accordance with the above XPS results. According to the Ni K‐edge EXAFS (Figure 2e), Co/Ni‐NC exhibits a main peak at 1.35 Å, similar to the Ni‐N scattering path of NiPc. The Ni‐N path was used to fit the first shell EXAFS of Co/Ni‐NC, and the fitted coordination number and bond distance are 4.1 and 1.83 Å (Figure S6b and Table S1, Supporting Information), respectively. In this regard, the Ni species in Co/Ni‐NC is mainly coordinated to four N atoms to form the atomic NiN_4_ sites.

Interestingly, the peak positions of both Co‐N and Ni‐N paths in Co/Ni‐NC have a slight negative shift, compared with CoPc and NiPc, as shown in the EXAFS spectra (Figures 2b,e). This finding was also supported by the wavelet transform (WT) of the EXAFS spectra (Figure 2f; Figure S7, Supporting Information). Accordingly, the Co K‐edge WT‐EXAFS contour plots of Co/Ni‐NC show a single‐intensity maximum at 4.0 Å^−1^ assigned to the Co─N bonds, which is very close to CoPc (4.2 Å^−1^). While the single‐intensity maximum of Ni‐N bonds in Co/Ni‐NC locates at 3.4 Å^−1^, much lower than that in NiPc (4.2 Å^−1^). Namely, unlike the symmetrical MN_4_ structure in CoPc and NiPc, the hetero‐diatomic metal pairs (CoN_4_‐NiN_4_) in Co/Ni‐NC involve local deformation (such as M‐N bond shortening) through long‐range electronic interaction, which is an alternative method for developing new high‐performance catalysts.^[^ ^23^, ^32^ ^]^

### 2.2 Electrochemical ORR/OER Performance

The bifunctional oxygen electrocatalysis performance of the prepared catalysts was systematically evaluated in alkaline solution. The benchmark Pt/C and RuO_2_ catalysts were also measured for comparison. All the potentials are provided versus reversible hydrogen electrode (RHE). In order to minimize the IR drop, the Ag/AgCl reference electrode was connected to the electrolyte through Luggin capillary. According to the electrochemical impedance spectra (EIS) shown in Figure S8 (Supporting Information), the Ohmic resistance of the electrolyte was significantly reduced from 53 to 6.8 Ω. In addition, the current for electrocatalytic oxygen reaction is relatively low (usually < 1 mA). Hance, this study's drop (<10 mV) is insignificant and acceptable.

**Figure 3:**
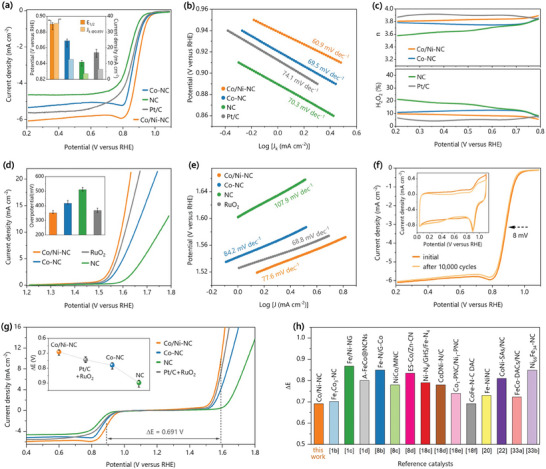
a) ORR LSV curves collected under 1600 rpm in O_2_ saturated 0.1 m KOH electrolyte (the insert is the corresponding half‐wave potential *E*_1/2_ and kinetic current density *J*_k_ at 0.85 V in the ORR LSV curves). b) The corresponding Tafel plots obtained by the ORR LSV curves. c) The electron transfer number (n, top) and H_2_O_2_ yield (%, bottom) in ORR process evaluated by RRDE method. d) OER polarization curves obtained in 0.1 m KOH electrolyte (the inset corresponds to the overpotential: *E*_j = 10_ – 1.23 V). e) Tafel plots obtained by the OER polarization curves. f) ORR LSV curves and CV curves (inset) for Co/Ni‐NC catalyst before and after 10 000 potential cycling between 0.6 and 1.0 V_RHE_ at 100 mV s^−1^. g) The combination of the ORR and OER polarization curves of different catalysts and the corresponding potential gap (Δ*E* = *E*_j = 10_ – *E*_1/2_, inset). h) Δ*E* comparison of the Co/Ni‐NC with the recently reported high‐performance bifunctional DACs.

Cyclic voltammetry (CV) tests in 0.1 m KOH solution were primarily performed, as presented in Figure S9 (Supporting Information). Accordingly, there is a significant oxygen reduction peak under O_2_‐saturated condition for Co/Ni‐NC peaked at 0.895 V, which is even more positive than Pt/C peaked at 0.862 V. Similar encouraging results were also obtained by linear sweep voltammetry (LSV) polarization test, as presented in **Figures** **3**a–c and S10a,b (Supporting Information). Among the measured catalysts, Co/Ni‐NC shows the best ORR catalytic performance, as evidenced by its superior capability to lower the ORR polarization barrier and accelerate the catalytic kinetics through the favorable 4‐electron pathway. Detailedly, the half‐wave potential (*E*_1/2_) and kinetic current density (*J*_k_) at 0.85 V in LSV curves are plotted and inserted in Figure 3a. As seen, Co/Ni‐NC shows the most positive *E*_1/2_ value of 0.890 V and the largest *J*_k_ value (35.5 mA cm^−2^, 0.85 V), superior to Pt/C (0.854 V and 6.4 mA cm^−2^ for *E*_1/2_ and *J*_k_ at 0.85 V, respectively). Tafel slopes derived from the ORR LSV curves are plotted in Figure 3b. Compared to other catalysts, Co/Ni‐NC exhibits the smallest Tafel slope, namely 60.9 mV dec^−1^, demonstrating its excellent catalytic kinetics for large *J*_k_ values. Moreover, the electron transfer number (n) and H_2_O_2_ yield in the ORR process were evaluated by rotating ring‐disk electrode (RRDE) tests, as shown in Figure 3c. The resulting n (>3.8) and H_2_O_2_ yield (<10%) catalyzed by Co/Ni‐NC reveal an almost ideal 4‐electron ORR pathway over the full potential range. The 4‐electron pathway is also supported by Koutecky‐Levich (K‐L) slopes shown in Figure S11 (Supporting Information), where the electron transfer number (n) of Co/Ni‐NC was calculated to be 3.91, very close to that of Pt/C (3.95).

As for OER performance, the prepared catalysts, and the benchmark RuO_2_ catalyst were also assessed by LSV polarization test in O_2_‐saturated 0.1 m KOH solution. The resulting LSV curves are plotted in Figure 3d and Figure S10c (Supporting Information), where the overpotential at 10 mA cm^−2^ (*E*_j_ *_=_*_10_ − 1.23 V, inset in Figure 3d) is used to evaluate the catalyst performance. Co/Ni‐NC exhibits the most negative onset potential and the smallest overpotential of 361 mV, indicating its highest OER catalytic activity, superior to RuO_2_ (377 mV) and other catalysts (>400 mV). The catalytic kinetics of the catalysts was assessed by the Tafel slopes derived from the LSV curves, as shown in Figure 3e and Figure S10d (Supporting Information). The Tafel slope of Co/Ni‐NC was determined to be 77.6 mV dec^−1^, close to RuO_2_ (68.8 mV dec^−1^), and superior to Co‐NC (84.2 mV dec^−1^) and NC (107.9 mV dec^−1^).

For long‐term durability tests, Co/Ni‐NC and Pt/C were cycled for 10 000 potential cycles from 0.6 to 1.0 V in an O_2_‐purged 0.1 m KOH solution. Co/Ni‐NC before and after the durability test shows similar CV and LSV behavior (Figure 3f), accompanied with only a slight loss in E_1/2_ (8 mV), which is much less significant than that of Pt/C (15 mV, Figure S12a, Supporting Information). Meanwhile, the durability tests caused only a slight increase in H_2_O_2_ yield and a slight decrease in electron transfer number for Co/Ni‐NC, which is much superior to Pt/C, as shown in Figure S12b (Supporting Information). These results highlight the excellent long‐term stability for Co/Ni‐NC against oxidation and degradation in ORR, even in comparison with Pt/C. Moreover, the long‐term OER stability was also evaluated by 2000 potential cycles from 1.2 to 1.6 V, as shown in Figure S13 (Supporting Information). After 2000 cycles, Co/Ni‐NC also exhibited very good OER stability, as evidenced by a positive shift of only 9 mV in *E*_j = 10_, much smaller than that of RuO_2_ (21 mV). TEM and XRD analyses (Figure S14, Supporting Information) were performed after the durability tests. In comparison with the pristine structure shown in Figures 1c,f,i,j, no significant difference was found for Co/Ni‐NC after both ORR and OER durability tests, implying its good operation stability.

LSV curves of ORR and OER were combined and shown in Figure 3g. Compared with the metal‐free NC catalyst, the loading of single‐atom metal active sites enhances both the ORR and OER activities for Ni‐NC (Figure S10, Supporting Information) and Co‐NC, especially for the latter. Furthermore, both the ORR and OER activities can be further improved by coupling the CoN_4_‐NiN_4_ pairs in Co/Ni‐NC, induced by long‐range synergistic effects between the paired sites (see Theoretical section below), although there is ≈0.41 nm intersite distance. The potential gap, denoted as Δ*E* (*E*_j_ *_=_*_10_ – *E*_1/2_), is usually applied to assess the performance of dual‐functional catalysts for OER and ORR. As calculated, the potential gap of Co/Ni‐NC is only 0.691 V, much lower than other tested catalysts, including the Pt/C‐RuO_2_ benchmark. Even in comparison with the recently reported high‐performance dual‐functional DACs (Δ*E*s in Figure 3h),^[^ ^1^, ^8^, ^18^, ^20^, ^22^, ^33^ ^]^ the Co/Ni‐NC catalyst remains robust and highly competitive for its excellent bifunctional oxygen electrocatalytic performance.

### 2.3 Zn–Air Battery Performance

**Figure 4:**
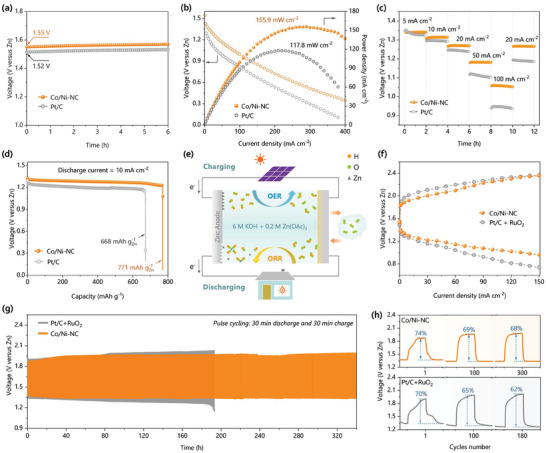
a) Open circuit voltages of Zn–air batteries with different cathode catalysts. b) Discharge polarization curves of Zn–air batteries under air atmosphere and the calculated power densities at different current densities. c) Rate performance of Zn–air batteries at different rate current densities. d) Discharge plots and the calculated specific capacity of Zn–air batteries at a rate of 10 mA cm^−2^. e) Schematic configuration of rechargeable Zn–air batteries. f) Charge and discharge polarization curves of rechargeable Zn–air batteries. g,h) Galvanostatic discharge‐charge cycling curves of the rechargeable liquid Zn–air batteries based on Co/Ni‐NC or Pt/C‐RuO_2_ catalysts.

The potential applications of the bifunctional Co/Ni‐NC catalyst were explored by assembling and evaluating Zn–air batteries and compared to the Pt/C benchmark. The electrolyte containing 6 m KOH and 0.2 m zinc acetate (dissolved to form Zn(OH)_4_^2−^) was used to ensure the reversible Zn anode reaction.^[^ ^34^ ^]^ The open circuit voltage of the Co/Ni‐NC‐based Zn–air battery was first recorded to be 1.55 V without clear decrease over 6 h, superior to Pt/C‐based Zn–air battery (1.52 V), as shown in **Figure** **4**a. The discharging polarization curves and power densities of the Zn–air batteries at different current densities are plotted in Figure 4b. Evidently, Co/Ni‐NC‐based battery always outperforms Pt/C‐based battery, in respect to both the cell voltage and power density, especially for their maximum power density (namely, 155.9 mW cm^−2^ at 290 mA cm^−2^ and 117.8 mW cm^−2^ at 210 mA cm^−2^ for Co/Ni‐NC and Pt/C respectively). Co/Ni‐NC also shows much better rate performance than Pt/C, as shown in Figure 4c. When the current density returned from 100 to 20 mA cm^−2^, the discharge voltage of Co/Ni‐NC‐based battery recovered rapidly with almost no deterioration, while Pt/C‐based battery suffered a sudden and serious voltage drop. Moreover, galvanostatic discharge curves were recorded at a current density of 10 mA cm^−2^, and the resultant specific capacities (normalized to the mass of the consumed Zn) were calculated, as shown in Figure 4d. Clearly, Co/Ni‐NC‐based battery could be discharged for a longer period at a higher voltage platform, resulting into a larger specific capacity of 771 mAh g^−1^, relative to Pt/C‐based battery (668 mAh g^−1^).

For rechargeable Zn–air battery (Figure 4e), the bifunctional Co/Ni‐NC catalyst was further investigated and compared with Pt/C‐RuO_2_ (1:1 by weight for bifunctionality). The charging and discharging polarization curves of the rechargeable Zn–air batteries are plotted in Figure 4f. Clearly, the voltage gap between the charging and discharging curves of Co/Ni‐NC‐based battery is always smaller than that of the battery based on Pt/C‐RuO_2_ catalyst, especially at high current density. For example, the voltage gap at 100 mA cm^−2^ is only 1.19 V for Co/Ni‐NC, much less than that for Pt/C‐RuO_2_ (1.41 V). This result is in good accordance with the efficient ORR and OER bifunctionality of Co/Ni‐NC, and implies less energy loss in practical application. Moreover, the cycling performance of the rechargeable batteries was tested at a current density of 2 mA cm^−2^ with a cycle duration of 60 min, as presented in Figures 4g,h. Accordingly, the battery catalyzed by Co/Ni‐NC can operate continuously for up to 340 cycles (h), with a small voltage gap of ≈0.65 V (the gap between discharge at ≈1.34 V and charge at ≈1.99 V) and a high round‐trip efficiency up to 74%. While Pt/C‐RuO_2_‐based battery could last only 180 cycles (h) with a large voltage gap of ≈0.76 V and a low round‐trip efficiency <70%. In comparison to the state‐of‐art rechargeable ZAB batteries listed in Table S2 (Supporting Information), Co/Ni‐NC‐catalyzed ZAB battery also shows competitive performance in terms of the open circuit voltage, peak power density, specific capacity, and long‐term durability. The excellent catalytic performance of Co/Ni‐NC for the rechargeable Zn–air batteries agrees well with its good ORR/OER bifunctionality of the atomically dispersed hetero‐diatomic metal sites with long‐range synergistic interaction.

### 2.4 Catalytic Mechanism by Theoretical Calculations

**Figure 5:**
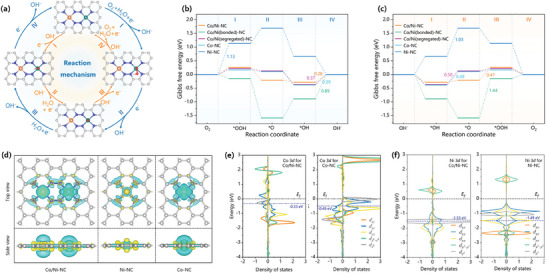
a) Optimized configurations for each elementary step on Co/Ni‐NC for ORR (clockwise) and OER (counter clockwise). Calculated free energy (ΔG) (in eV) profiles for b) ORR and c) OER processes on catalysts under alkaline condition at U = 1.23 V. d) Charge density difference of Co/Ni‐NC, Co‐NC, and Ni‐NC (iso‐surface = 0.008 a.u., yellow and blue regions represent electron accumulation and electron depletion, respectively). Partial density of states of Co 3d e) for Co/Ni‐NC (left) and Co‐NC (right) and Ni 3d f) for Co/Ni‐NC (left) and Ni‐NC (right).

Density functional theory (DFT) calculations were performed to further explore the underlying mechanism of the bifunctional Co/Ni‐NC catalyst. Co‐NC, Ni‐NC and the directly bonded and absolutely segregated CoN_4_ and NiN_4_ sites (Co/Ni(bonded)‐NC, *d*_Co‐Ni_ = 2.32 Å; Co/Ni(segregated)‐NC, *d*_Co‐Ni_ = 8.44 Å, see schematic in Figure S15, Supporting Information) were also calculated for comparison, as shown in **Figure** **5**, and Figures S16–S19 and Tables S3 and S4 (Supporting Information). According to the free energy profiles of the ORR and OER reactions (Figure 5a–c), the potential determining steps for the Co/Ni‐NC‐catalyzed ORR and OER reactions were calculated to be the *OH desorption and *OOH formation, respectively, differing from other catalysts. Moreover, the ORR/OER overpotentials generally increase in the order of Co/Ni‐NC (0.28/0.47 eV) < Co‐NC (0.39/0.49 eV) ≈ Ni/Co(segregated)‐NC (0.37/0.50 eV) < Ni‐NC (1.13/1.03 eV) ≈ Ni/Co(bonded)‐NC (0.89/1.44 eV). In this order, Co/Ni‐NC shows the lowest overpotentials for both the ORR and OER reactions, consistent with its best bifunctional activity among the studied catalysts. The order is also consistent with the experimental results (ORR: *E*_1/2_, V; OER: *E*_j_ *_=_*_10_ − 1.23 V, mV), where the catalytic activities decrease in the order of Ni/Co‐NC (0.890 V, 361 mV) > Co‐NC (0.868 V, 410 mV) ≈ Co/Ni(30%)‐NC (0.861 V, 413 mV) > Ni‐NC (0.844 V, 441 mV) ≈ Co/Ni(300%)‐NC (0.851 V, 438 mV) (Co/Ni(300%)‐NC and Co/Ni(30%)‐NC roughly stand for Ni/Co(bonded)‐NC and Ni/Co(segregated)‐NC, respectively, see detail in Figure S20, Supporting Information). According to the adsorption energies (AE) of the intermediates listed in Table S3 (Supporting Information), in Ni/Co‐NC the AE values on the Co site (−1.39 to −3.67 eV) are moderate and suitable for high activity,^[^ ^35^ ^]^ while the adsorption energies on the Co site (−0.76 to −2.54 eV) are relatively low, suggesting that the Co site in Co/Ni‐NC is the active site for both the ORR and OER reactions. The moderate AE values were also found for Co‐NC (−1.51 to −3.33 eV) and Co/Ni(segregated)‐NC (−1.43 to −3.30 eV) with fair catalytic activity, and the AE values for the poor catalysts are either too low (−0.51 to −1.74 eV, Ni‐NC) or too high (−1.80 to −5.08 eV, Co/Ni(bonded)‐NC). These results demonstrate the positive effect of the NiN_4_ site on improving the bifunctional ORR/OER activity of its adjacent CoN_4_ active site through electrical interaction.

The synergistic effect of CoN_4_‐NiN_4_ pairs on ORR and OER catalytic activities was further analyzed by electron configuration. According to the charge density difference (Figure 5d) and the Bader charges of Ni and Co (Table S4, Supporting Information), there is significant electron delocalization from metals to their adjacent N atoms, making the Bader charges range from +0.82 to +0.85 |e|, except Co/Ni(bonded)‐NC (+0.56 to +0.65 |e|). Such electron delocalization is conducive to the adsorption of O_2_ for improving the catalytic activity (Figures S16–S18, Supporting Information). The metal electrons of Co/Ni(bonded)‐NC appear to be localized between the Co─Ni bond for low Bader charges and poor catalytic activity. Similarly, Searles et. al.^[^ ^36^ ^]^ used DFT calculations to demonstrate that the ORR overpotential of CoNi@N6V4 (0.87 V, *d*_Co‐Ni_ = 2.1 Å) is far higher than that of CoNi@N8V4 (0.35 V, *d*_Co‐Ni_ = 4.1 Å). According to the charge density difference and partial density of states depicted in Figure 5d–f and Figure S19 (Supporting Information), the *d* band center of Co in Co/Ni‐NC (versus that in Co‐NC) obviously moves toward the Fermi level, and the *d* band center of Ni (versus that in Ni‐NC) moves away from the Fermi level, achieving *d*–*d* coupling between Co and Ni. It can be stated that the long‐range *d‐*‐*d* coupling between the CoN_4_‐NiN_4_ pair modulates the electron configuration and the intermediates’ affinity of the CoN_4_ active site for its excellent electrocatalytic oxygen reaction activity.^[^ ^37^ ^]^

## 3 Conclusion

In conclusion, hetero‐diatomic site pairs (CoN_4_‐NiN_4_) were uniformly dispersed on the nitrogen‐doped carbon matrix by the controlled pyrolysis of ZIF‐8 containing Co^2+^ and Ni^2+^ species. The resulting Co/Ni‐NC catalyst was systematically characterized by SEM, TEM, EDS, XRD, XPS, and XAS for identifying the CoN_4_‐NiN_4_ configuration with an intersite distance ≈0.41 nm, supported by theoretical calculations. Carbon nanotubes (≈20 nm in diameter) at the edges of the carbon particles were also detected for high conductivity for electrocatalysis. Co/Ni‐NC was tested to have superior ORR and OER bifunctionality with a potential gap of only 0.691 V and long‐term stability, much better than the single‐atom Co‐NC and Ni‐NC catalysts and the benchmark Pt/C and RuO_2_ catalysts. Therefore Co/Ni‐NC was used as air electrode catalyst for Zn–air batteries, which have a high capacity up to 771 mAh g^−1^ and can operate continuously for up to 340 h with a small voltage gap of only ≈0.65 V, also superior to Pt/C‐RuO_2_. Theoretical calculations reveal that the superior bifunctionality of the hetero‐diatomic CoN_4_‐NiN_4_ pairs should be related to the *d*–*d* orbital coupling between Co and Ni and the metallic electron delocalization for improving the O_2_‐affinity of the Co sites.

## Conflict of Interest

The authors declare no conflict of interest.

## Supporting information

Supporting Information

## Data Availability

The data that support the findings of this study are available from the corresponding author upon reasonable request.
