# Supplementary material for: Hetero‐Diatomic CoN4‐NiN4 Site Pairs with Long‐Range Coupling as Efficient Bifunctional Catalyst for Rechargeable Zn–Air Batteries
Source: Adv Sci (Weinh). 2024 Mar 30;11(22):2310231. doi: 10.1002/advs.202310231 (PMC11165470; doi:10.1002/advs.202310231)
Supplement: Supplementary file 1 — Supporting Information [file ADVS-11-2310231-s001.pdf]

ADVANCED SCIENCE
Open Access

Supporting Information

for *Adv. Sci.*, DOI 10.1002/advs.202310231

Hetero-Diatomic CoN_4-NiN_4 Site Pairs with Long-Range Coupling as Efficient Bifunctional Catalyst for Rechargeable Zn–Air Batteries

Yue Yang, Bin Li, Yining Liang, Wenpeng Ni, Xuan Li, Gengzhe Shen, Lin Xu, Zhengjian Chen, Chun Zhu, Jin-Xia Liang* and Shiguo Zhang**

Hetero-Diatomic CoN_4-NiN_4 Site Pairs with Long-Range Coupling as Efficient Bifunctional Catalyst for Rechargeable Zn-Air Batteries

Yue Yang [a,†], Bin Li [b,†], Yining Liang [a], Wenpeng Ni [c], Xuan Li [a], Gengzhe Shen [a], Lin Xu [a], Zhengjian Chen [a,*], Chun Zhu [b], Jin-Xia Liang [b,*], Shiguo Zhang [c,*]

[a] Zhuhai Institute of Advanced Technology, Shenzhen Institutes of Advanced Technology, Chinese Academy of Sciences, Zhuhai 519000, China.

[b] School of Chemistry and Chemical Engineering, Guizhou University, Guiyang 550025, China.

[c] College of Materials Science and Engineering, State Key Laboratory of Advanced Design and Manufacturing for Vehicle Body, Hunan University, Changsha 410004, China.

† *These authors contributed equally to this work.*

* Corresponding author: chenzhengjian@ziat.ac.cn (Z. Chen); jxliang@gzu.edu.cn (J.-X Liang); zhangsg@hnu.edu.cn (S. Zhang).

Supporting Information

1. Experimental

1.1 Chemicals

Cobalt chloride hexahydrate (99%, aladdin), Nickel chloride hexahydrate (99%, aladdin), zinc acetate (99%, aladdin), 2-methylimidazole (99%, J&K Scientific), ethylenediaminetetraacetic acid (EDTA, 98%, J&K Scientific), Nafion dispersion (5 wt.%, Alfa Aesar), Pt/C (20 wt.% metal, Alfa Aesar), RuO_2 (99.9%, Strem), methanol (99.5%, Sinopharm Chemical), KOH (99.99%, Alfa Aesar) and hydrophobic carbon cloth (W1S1009, CeTech) were used as received without further treatment. Ultrapure

water with the specific resistance of 18.2 MΩ·cm was obtained by reversed osmosis followed by ion-exchange and filtration.

1.2 Material synthesis

Zeolite imidazolate framework-8 (ZIF-8): 8 mmol $Zn(CH_3COO)_2$ and 40 μl HCl (37% solution) were dissolved in 80 ml ultrapure water to form a clear solution, which was injected into 80 ml ultrapure water containing 64 mmol 2-methylimidazole and then continuously stirred for 8 h at room temperature. After centrifuging and washing with ethanol and drying in vacuum at 70 °C for overnight, milky white hierarchical porous ZIF-8 product was obtained.

CoNi-ZIF-8: 80 mg EDTA, $CoCl_2 \cdot 6H_2O$ (0.32 mmol), $NiCl_2 \cdot 6H_2O$ (4.0 mmol), $Zn(CH_3COO)_2 \cdot 6H_2O$ (8.0 mmol) and 40 μl HCl (37% solution) were dissolved in 80 ml ultrapure water to form a clear solution, which was injected into 80 ml ultrapure water containing 5.26 g 2-methylimidazole. After continuous stirring for 8 h at room temperature, resulting precipitate was centrifuged, washed with ethanol and dried in vacuum at 70 °C for overnight to generate the CoNi-ZIF-8 product. Co-ZIF-8 and Ni-ZIF-8 was also synthesized following the procedure used for CoNi-ZIF-8 except that no $NiCl_2 \cdot 6H_2O$ and $CoCl_2 \cdot 6H_2O$ was added, respectively.

Co/Ni-NC, Co-NC, Ni-NC and NC carbon materials: In a typical procedure, the powder of CoNi-ZIF-8 was placed in a tube furnace and then heated to 950 °C at a heating rate of 5 °C/min and finally naturally cooled down to room temperature under flowing argon gas. The Co/Ni-NC catalyst was obtained as black powder and directly used without any post-treatment.

1.3 Characterizations

Scanning electron microscope (SEM) images were recorded on a Czech TESCAN MIRA LMS SEM operated at 3 kV and equipped with a SE2 detector. Transmission electron microscope (TEM) images were performed using a Thermo Scientific Talos F200 operating at 200 kV. Energy dispersive X-ray spectroscopy (EDS) mappings were collected under high-angle annular dark-field scanning transmission electron microscopy (HAADF-STEM) mode. The atomic-resolution microscopy analysis was performed on a JEOL JEM-ARM200F microscope equipped with a probe spherical aberration (Cs) corrector at 200 kV. The specific surface area (m^2 g^{-1}) and pore size distribution were determined by a JW-BK200C BET analyzer through N_2 adsorption-desorption isotherms. X-ray Diffraction (XRD) patterns were obtained on a Rigaku Ultima IV diffractometer equipped with monochromatic Cu Kα source (λ=1.5418Å) at 40 kV and 150 mA. X-ray photoelectron spectroscopy (XPS) was measured using a Thermo Scientific K-Alpha XPS system equipped with an Al Kα micro-focused monochromatic source (1486.6 eV). The XPS binding energies were calibrated by C 1s peak at 284.8 eV. Raman spectra were investigated using a WiTec alpha300R high resolution confocal Raman spectrometer. Inductively coupled plasma mass spectra (ICP-MS) were performed on an Agilent ICP-MS 7700 instrument.

Extended X-ray absorption fine structure spectra (EXAFS, Co and Ni K-edge) were collected at 4B9A beamline in Beijing Synchrotron Radiation Facility (BSRF) under ambient conditions. The storage rings of BSRF were operated at 2.5 GeV with a stable current of 400 mA. Using Si (111) double-crystal monochromator, the data collection

was carried out in transmission mode. Data reduction, data analysis, and EXAFS fitting were performed and analyzed with the Athena and Artemis programs of the Demeter data analysis packages that utilizes the FEFF6 program to fit the EXAFS data.[1] The energy calibration of the sample was conducted through standard Co foil and Ni foil, which as a reference was simultaneously measured. A linear function was subtracted from the pre-edge region, then the edge jump was normalized using Athena software. The $\chi(k)$ data were isolated by subtracting a smooth, third-order polynomial approximating the absorption background of an isolated atom. The *k3*-weighted $\chi(k)$ data were Fourier transformed after applying a Hanning window function (Δk = 1.0). For EXAFS modeling, The global amplitude EXAFS (*CN, R,* σ^2 and ΔE_0) were obtained by nonlinear fitting, with least-squares refinement, of the EXAFS equation to the Fourier-transformed data in *R*-space, using Artemis software, EXAFS of the Co foil and Ni foil are fitted and the obtained amplitude reduction factor S_0^2 value (0.885 and 0.887) was set in the EXAFS analysis to determine the coordination numbers (*CNs*) in the Co/Ni-N/Co scattering path in sample. For Wavelet Transform analysis, the $\chi(k)$ exported from Athena was imported into the Hama Fortran code.[2] The parameters were listed as follow: *R*-range, 1-3.5 Å, *k*-range, 0-13.0 $Å^{-1}$ for sample (0-13.0 $Å^{-1}$ for Co foil, Ni foil, CoO, CoPc, Co_3O_4, NiO and NiPc); *k* weight, 2; and Morlet function with κ=9, σ=1 was used as the mother wavelet to provide the overall distribution.

1.4 Electrochemical measurements

5 mg of catalyst and 20 μl 5 wt.% Nafion solution were dispersed in 1 ml 3:1 v/v ultrapure water/isopropanol by sonication for at least 60 min to form a homogeneous

ink. Then 15 μl ink was loaded onto a glassy carbon rotating disk electrode (RDE). The final loading for all the electrocatalysts on the RDE electrode was ~0.6 mg cm^{-2}. Electrochemical tests were performed in a three-electrode system (CHI 760E) with a catalyst-loaded RDE working electrode, a graphitic carbon rod counter electrode and an Ag/AgCl (3 M KCl solution) reference electrode. All the potentials were referred to reversible hydrogen electrode (RHE) by the following calculations:

$$E_{(RHE)} = E_{(Ag/AgCl)} + 0.0591 \cdot pH + 0.210\ (V)$$

The ORR tests by cyclic voltammetry (CV) and linear sweep voltammetry (LSV) were performed in O_2 saturated 0.1 M KOH solution from 1.0 to 0.2 V_{RHE} at a scan rate of 5 mV s^{-1} and various rotating speeds from 400 to 2025 rpm. The OER tests were operated between 1.0 and 1.8 V_{RHE} at a scan rate of 5 mV s^{-1} with a rotating speed of 1600 rpm to ease the diffusion of evolved oxygen. In the ORR polarization curves, the current densities were normalized in reference to the geometric area of the RDE (0.125 cm^2). Koutecky-Levich (K-L) plots were derived from LSV curves at the applied electrode potentials. Electron transfer number (n) per O_2 molecule involved in ORR process was calculated by Levich equation as shown in Equation (1):

$$\frac{1}{J} = \frac{1}{J_l} + \frac{1}{J_K} = \frac{1}{B\omega^{1/2}} + \frac{1}{J_K} \quad (1)$$

where J is the measured current density, J_K and J_L are the kinetic and diffusion limiting current densities respectively, ω is the electrode rotating speed (rpm). B can be calculated from the Koutecky equation as shown in Equation (2):

$$B = 0.62\mathrm{nF}D_o^{2/3}C_o\nu^{-1/6} \quad (2)$$

where F is Faraday constant (96485 C mol^{-1}), D_O is the diffusion coefficient of O_2 in 0.1 M KOH solution (1.9×10^{-5} cm^2 s^{-1}), ν is the kinetic viscosity (0.01013 cm^2 s^{-1}), and C_O corresponds to the concentration of O_2 in the electrolyte (1.2×10^{-6} mol cm^{-3}). The catalysts loaded on a rotating ring-disk electrode (RRDE) composed of a glassy carbon disk (5 mm diameter) and a Pt ring were also conducted to evaluate their four-electron selectivity in the ORR process. The ring electrode was set at a constant potential of 1.4 V_{RHE} to detect the as-generated HO_2^- intermediate. Electron transfer number (n) and hydrogen peroxide yield (H_2O_2%) were calculated by the following equations (3) and (4), respectively:

$$n = 4 \times \frac{j_d}{j_d + \frac{j_r}{N}} \tag{3}$$

$$HO_2^- = 200 \times \frac{\frac{j_r}{N}}{j_d + \frac{j_r}{N}} \ (\%) \tag{4}$$

Where N (37%) is the collection efficiency of the ring electrode, j_r is the ring current and j_d is the disk current. The ORR durability was performed in O_2 saturated 0.1 M KOH solution by applying the cyclic potential sweeps between 0.6 and 1.0 V_{RHE} at a rate of 100 mV s^{-1} for 10,000 cycles. The OER durability was also evaluated by potential cycling 0.1 M NaOH solution for 2,000 cycles in the potential range from 1.2 to 1.6 V_{RHE} at a rate of 50 mV s^{-1}.

1.5 Assembly and test of Zn-air batteries

The Co/Ni-NC catalyst was coated on hydrophobic carbon cloth (~1.2 mg cm^{-2}) to form an air cathode for assembling Zn-air batteries. The surface area of the cathode

exposed to air was 1 cm^2. The polished Zn plate with a thickness of 0.3 mm was used as anode, and 6 M KOH aqueous solution containing 0.2 M $Zn(CH_3COO)_2$ was used as electrolyte. For comparison, the commercial Pt/C and Pt/C-RuO_2 (1:1, mass ratio) were used for assembling primary and rechargeable Zn-air batteries, respectively. The home-made Zn-air batteries were evaluated on a Neware battery testing system. The specific capacity (C_m) was determined at a discharge current density of 10 mA cm^{-2} and was normalized to the mass of Zn, according to the following equation:

$$C_m(\text{mAh g}^{-1}) = \frac{\text{discharge current (mA)} \times \text{time (h)}}{\text{weight of consumed Zn (g)}} \tag{5}$$

2. Density functional computational

All calculations were performed using Vienna Ab initio Simulation Package (VASP).[3] The electronic exchange-correlation potential was described by the generalized gradient approximation (GGA) with the PBE functional.[4] The projector-augmented wave (PAW) method was used to describe the electron-ion interaction, and the plane-wave basis was cut off by 500 eV. The dispersion correction by Grimme's method (DFT-D3) was used to correct the Van der Waals interaction.[5] All geometric models were constructed based on the graphene 8×8 supercell. The conjugated gradient method with a converging tolerance of 0.02 eV/Å for the force on each atom was used for the full geometry optimization without any restrictions. Periodic boundary conditions were considered along with the growth directions of the 2D materials, with a vacuum distance of 20 Å to avoid artificial interlay interaction between the adjacent units of the periodic 2D materials. A 1×1×1 Monkhorst-Pack grid was adopted for

geometry optimization, while 5×5×1 Monkhorst-Pack grid was used for the partial density of states (PDOS).[6] The processes of four-electron ORR and OER at the surface of Co/Ni-NC is as follows. In an alkaline electrolyte, the ORR can be decomposed into the following elementary steps:

$$* + H_2O(l) + O_2(g) + e^- \rightarrow OOH^* + OH^- \tag{6}$$

$$OOH^* + e^- \rightarrow O^* + OH^- \tag{7}$$

$$O^* + H_2O(l) + e^- \rightarrow OH^* + OH^- \tag{8}$$

$$OH^* + e^- \rightarrow * + OH^- \tag{9}$$

The OER process can be regarded as the inverse of the ORR process, its proceeds via the four-electron-transfer pathway as shown below:

$$* + OH^- \rightarrow OH^* + e^- \tag{10}$$

$$OH^* + OH^- \rightarrow O^* + H_2O(l) + e^- \tag{11}$$

$$O^* + OH^- \rightarrow OOH^* + e^- \tag{12}$$

$$OOH^* + OH^- \rightarrow * + H_2O(l) + O_2(g) + e^- \tag{13}$$

where * represents the active centers.

The adsorption energy (E_{ads}) of an adsorbate (X) on the adsorbent Co/Ni-NC was calculated according to the following equation:

$$E_{\mathrm{ads}} = E_{\mathrm{X^*Co/Ni-NC}} - E_{\mathrm{Co/Ni-NC}} - E_{\mathrm{X}} \tag{14}$$

where $E_{\mathrm{X^*Ni/Co-NC}}$, $E_{\mathrm{Ni/Co-NC}}$ and E_{X} represent the total energy of the adsorption system, substrate, and adsorbates, respectively.

Using the computational hydrogen electrode (CHE) model,[7] the Gibbs free energy change of each elementary step of ORR and OER is defined as:

$$\Delta G = \Delta E_{\mathrm{DFT}} + \Delta E_{\Delta \mathrm{ZPE}} - T\Delta S + \Delta G_{\mathrm{U}} + \Delta G_{\mathrm{pH}} \quad (15)$$

$$\Delta G_{\mathrm{pH}} = 2.303 k_{\mathrm{B}} T \,\mathrm{pH} \quad (16)$$

where ΔE_{DFT} is the reaction energy obtained from the DFT calculations; $\Delta E_{\Delta \mathrm{ZPE}}$ is the difference of zero-point energy of the reactants and products; T is the temperature (T=298.15 K); ΔS is the difference of entropy; $\Delta G_{\mathrm{U}} = -neU$, where n is the number of electrons transferred, and U is the electrode potential; ΔG_{pH} is the free energy correction of pH, and the pH is assumed to be 14 for the alkaline conditions in this work.

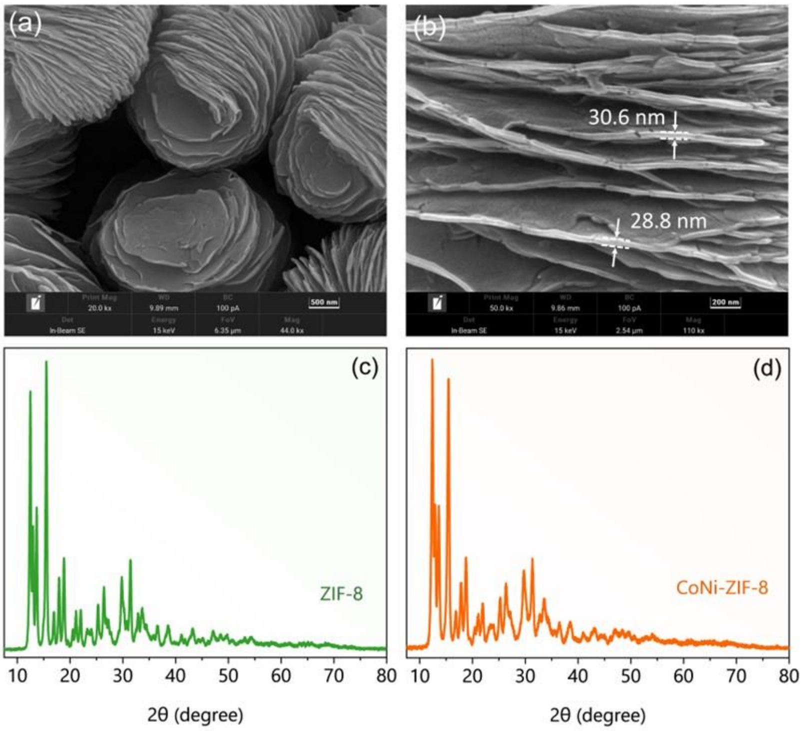

Figure S1. (a,b) SEM images of CoNi-ZIF-8. XRD patterns of (c) ZIF-8 and (d) CoNi-ZIF-8.

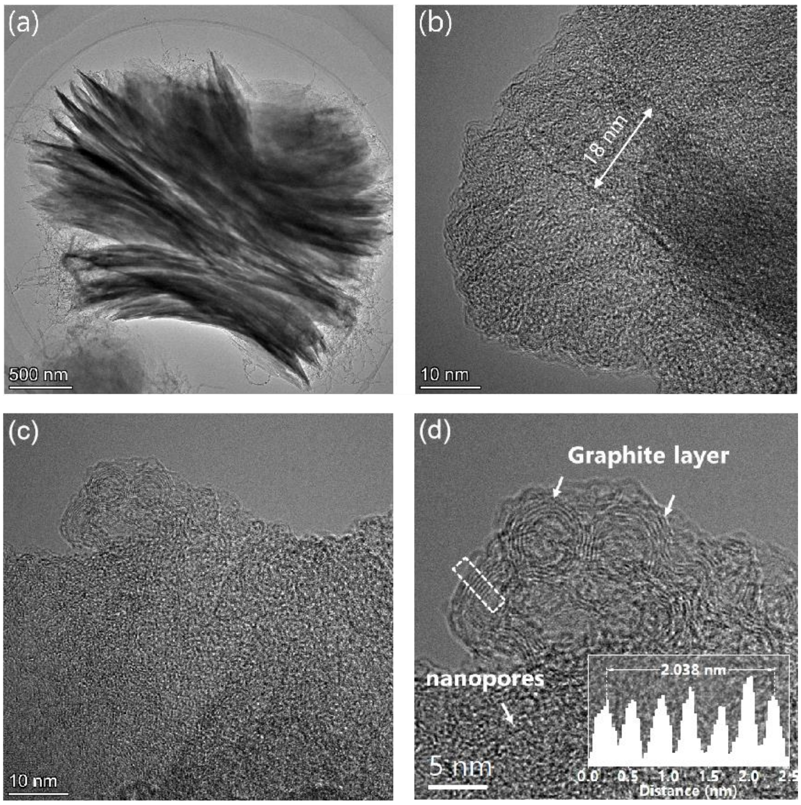

Figure S2. TEM images of (a) the Co/Ni-NC particle with carbon nanotubes at the edge and (b) carbon nanotubes grown on the surface. (c,d) HRTEM images for the observation of graphite layers and nanopores.

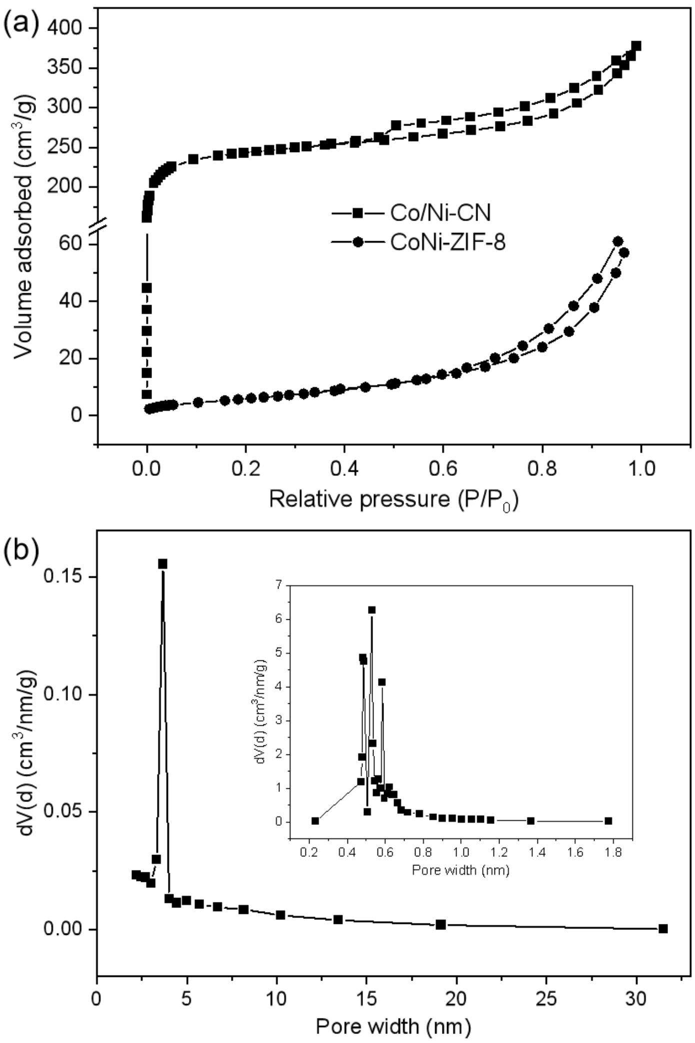

Figure S3. (a) N_2 adsorption-desorption isotherms of Co/Ni-NC (734 cm^2 g^{-1}) and CoNi-ZIF-8 (24.9 cm^2 g^{-1}). (b) Pore size distribution of Co/Ni-NC (inset shows the detail of the micropore region).

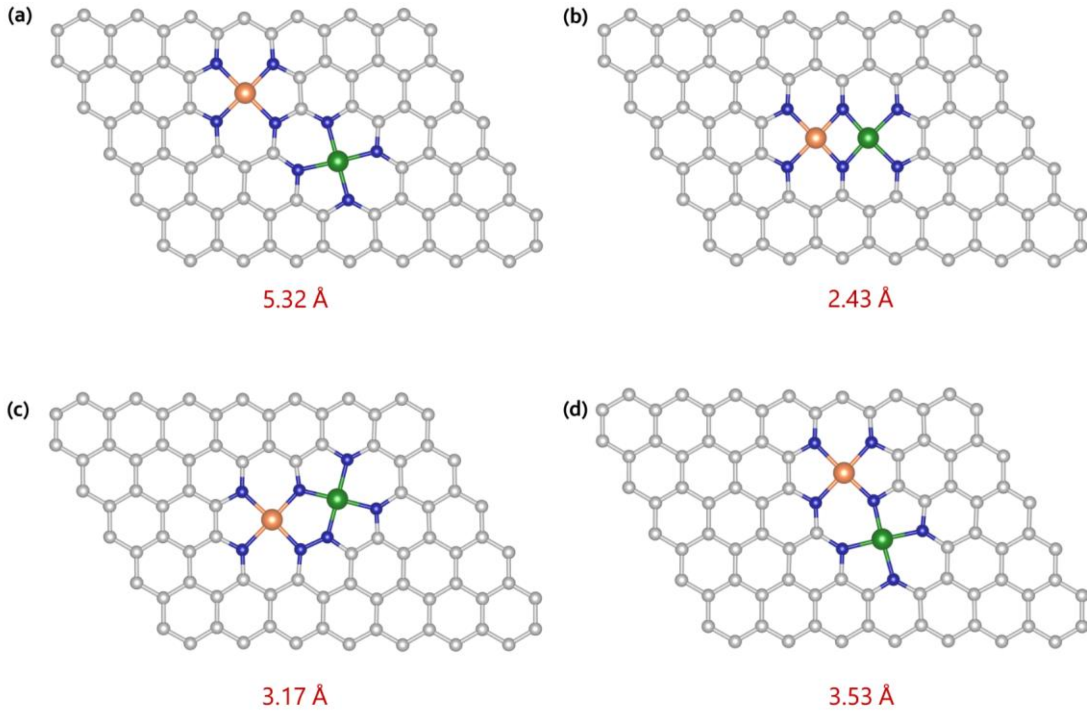

Figure S4. The schematic models of adjacent Co-N_4 and Ni-N_4 sites and the distances between Co and Ni atoms determined by theoretical calculations (the orange, green, blue and gray spheres represent Ni, Co, N and C atoms, respectively).

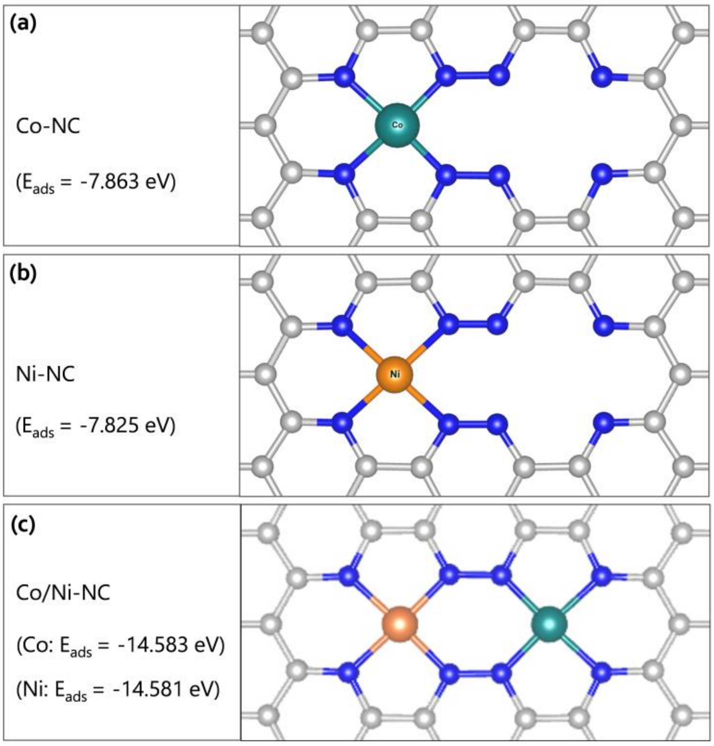

Figure S5. The adsorption energy (E_{ads}) of metals at defects in nitrogen-doped carbon: (a) Co-NC, (b) Ni-NC and (c) Co/Ni-NC (orange: Ni; green: Co; blue: N; gray: C).

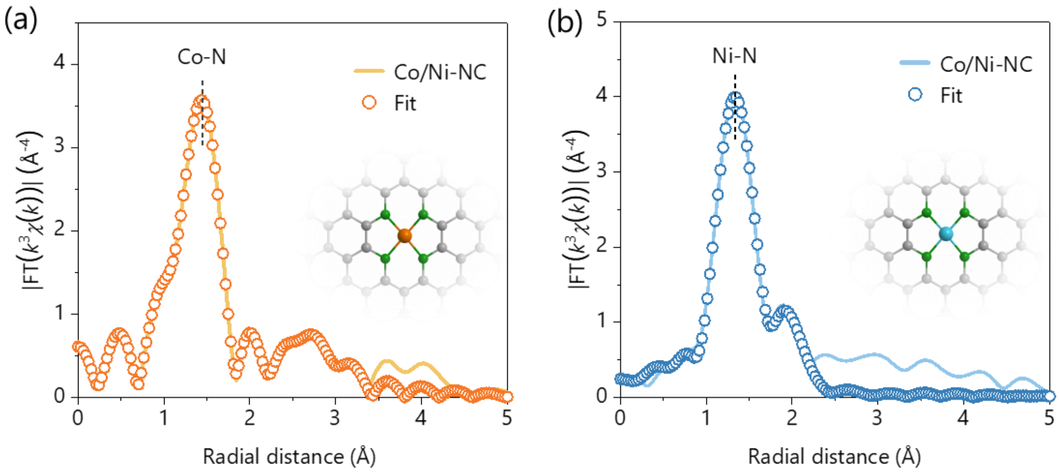

Figure S6. Fitting results of (a) Co K-edge and (b) Ni K-edge FT-EXAFS for Co/Ni-NC.

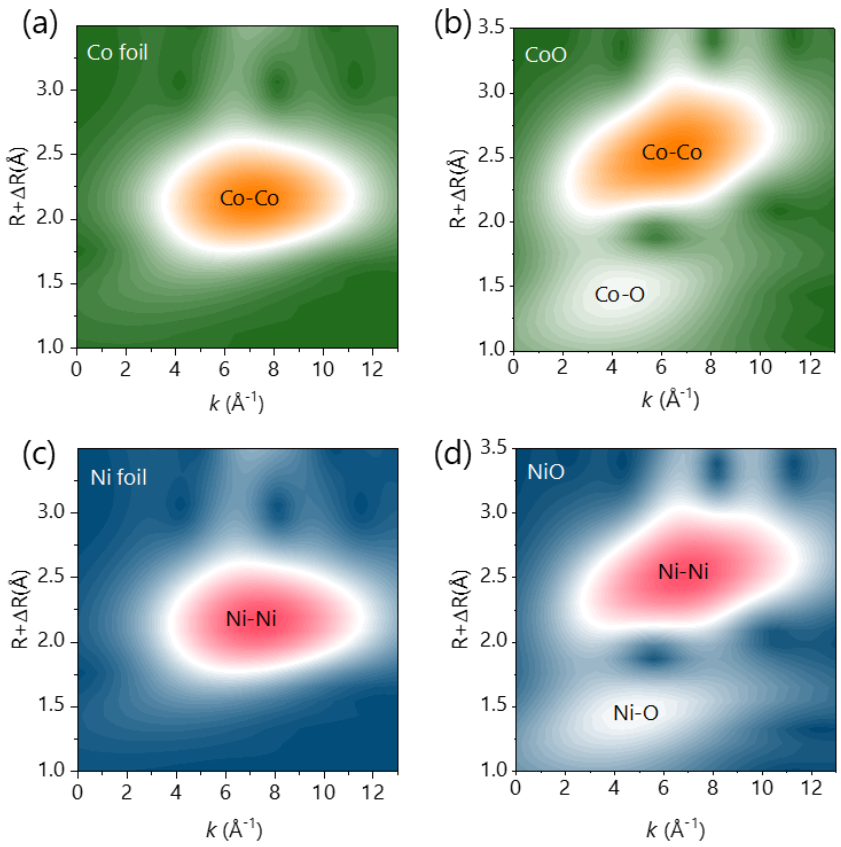

Figure S7. Wavelet transform of the k^3-weighted EXAFS data of (a) Co foil, (b) the Co K-edge of CoO, (c) Ni foil, and (d) the Ni K-edge of NiO.

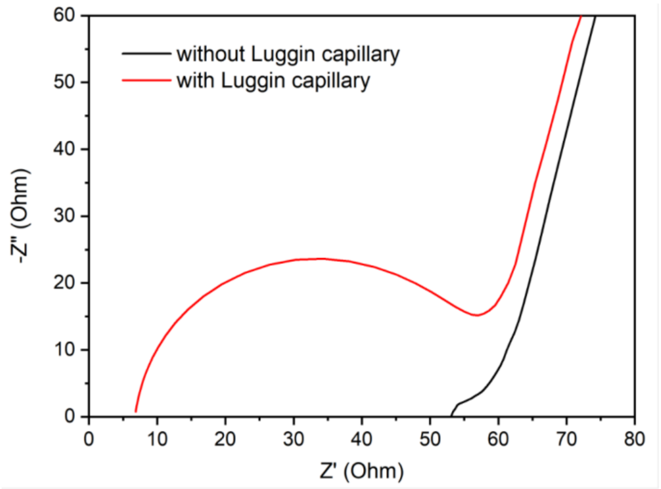

Figure S8. Nyquist plots of EIS of the three-electrode system (Co/Ni-NC-loaded RDE working electrode, graphitic carbon rod counter electrode and Ag/AgCl reference electrode, performed in 0.1 M KOH electrolyte using a CHI760E electrochemical workstation with an amplitude of 10 mV and a frequency from 0.01 Hz to 1 MHz).

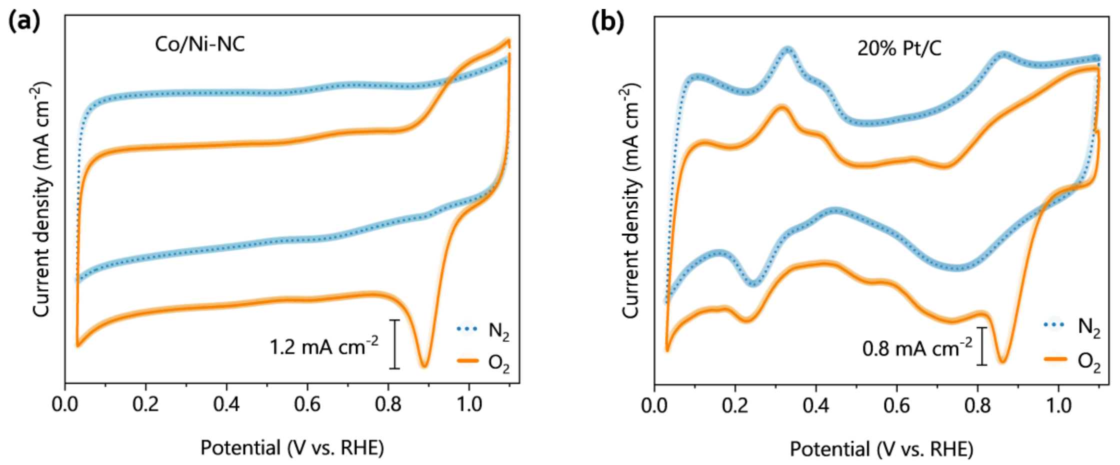

Figure S9. (a) CV curves of (a) Co/Ni-NC and (b) Pt/C in 0.1 M KOH electrolyte.

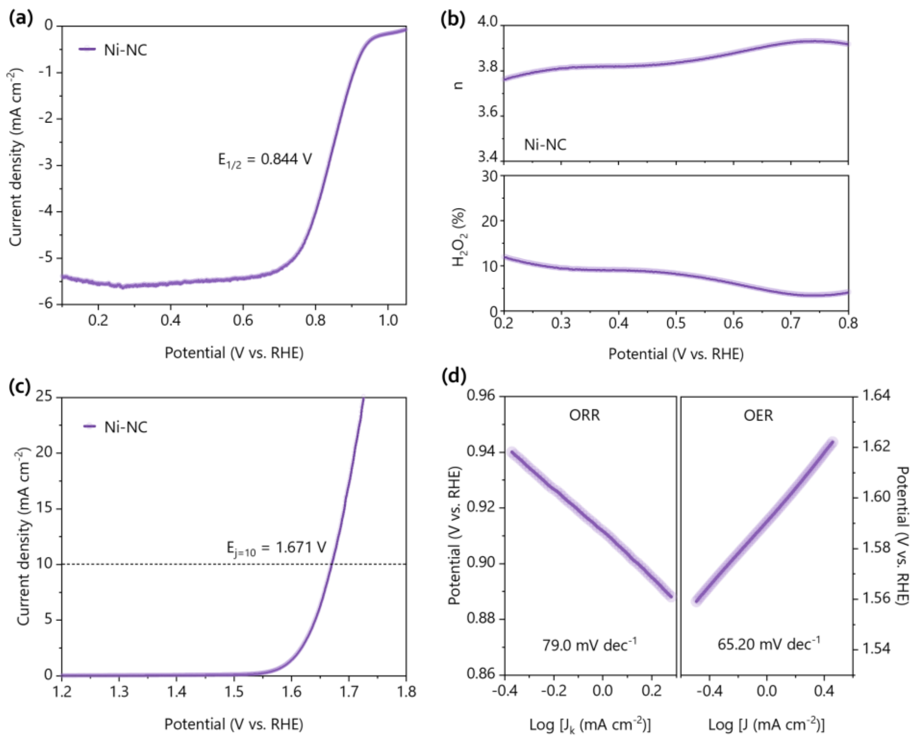

Figure S10. (a) ORR polarization curve of Ni-NC in O_2 saturated 0.1 M KOH electrolyte and (b) the corresponding electron-transfer number (n, top) and H_2O_2 yield (%, bottom) at 0.2 to 0.8 V_{RHE}, (c) OER polarization curve of Ni-NC in 0.1 M KOH electrolyte, (d) Tafel plots obtained by the ORR and OER polarization curves of Ni-NC, respectively.

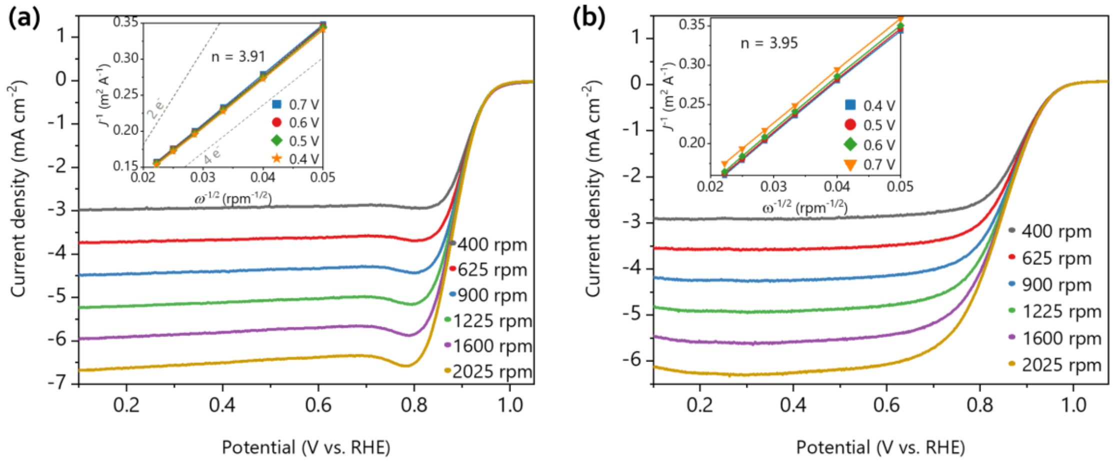

Figure S11. RDE polarization profiles during ORR for (a) Co/Ni-NC and (b) Pt/C with different sweep rates (rpm). Insert chart: characteristic K-L plots at different potentials including transferred electron number (n).

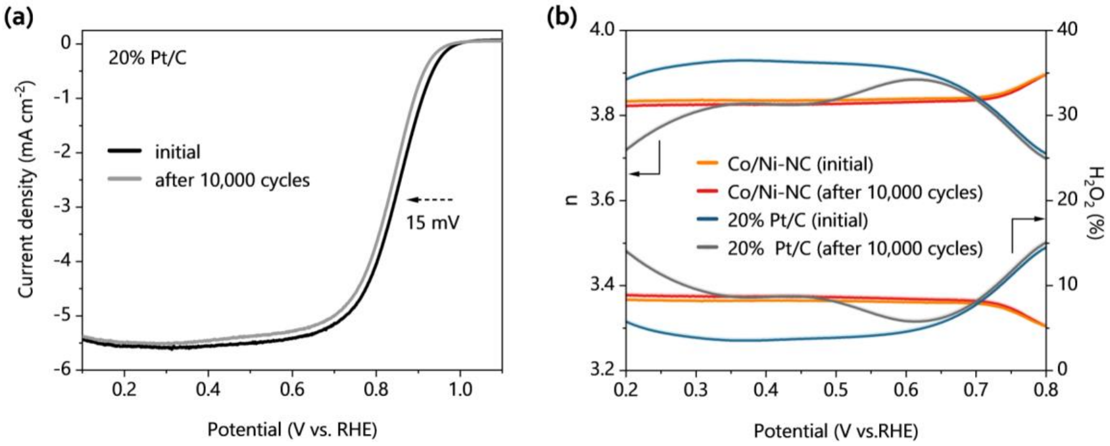

Figure S12. (a) ORR polarization curves of Pt/C catalyst and (b) electron transfer number (n) and H_2O_2 yield of Co/Ni-NC and Pt/C before and after 10,000 potential cycling between 0.6 and 1.0 V_{RHE} at a rate of 100 mV s^{-1}.

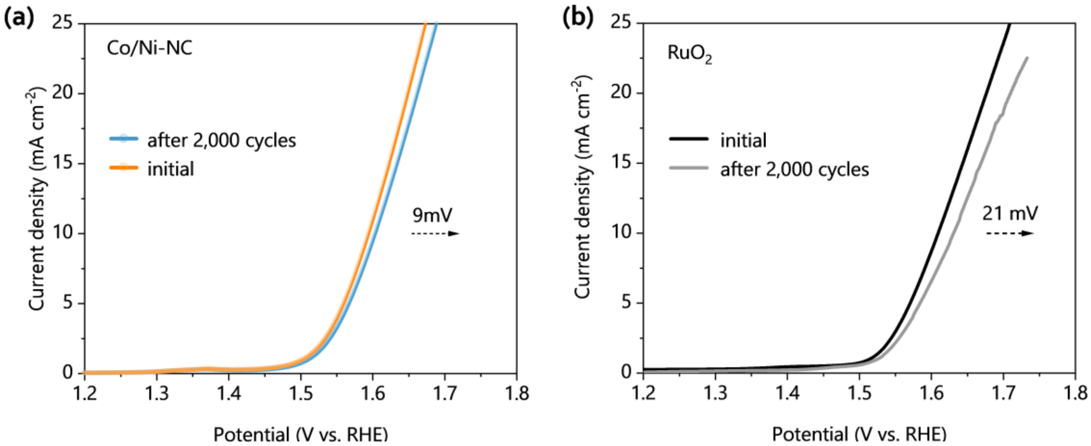

Figure S13. OER polarization curves of (a) Co/Ni-NC and (b) RuO_2 before and after 2,000 potential cycles (1.2-1.6 V_{RHE}, 50 mV s^{-1}).

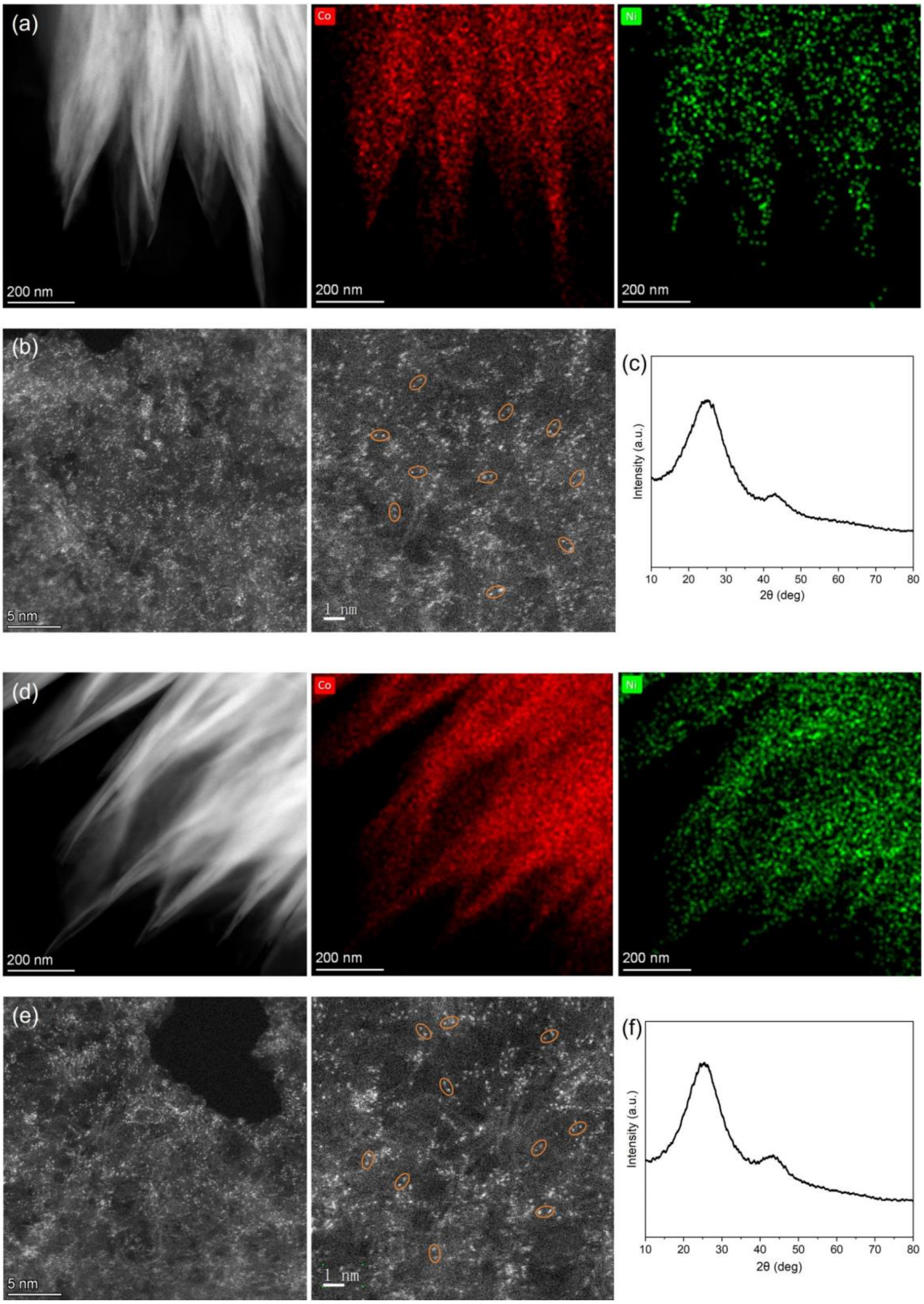

Figure S14. (a, d) EDS mappings for Co and Ni elements, (b, e) Aberration-corrected HAADF-STEM images, and (c, f) XRD spectra of Co/Ni-NC after long-term ORR (upper) and OER (bottom) durability tests.

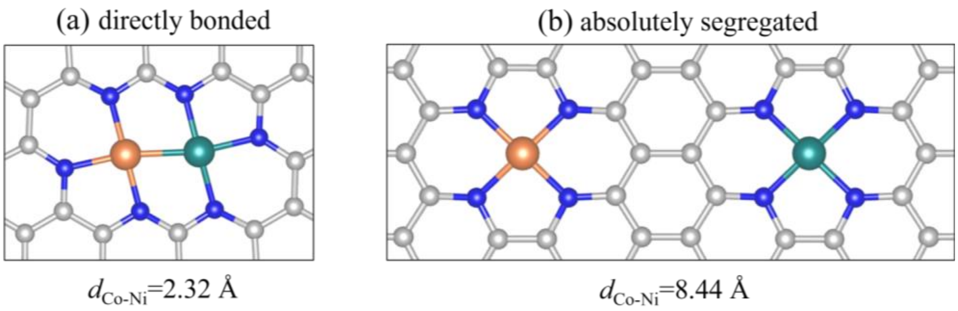

Figure S15. Schematic model of directly bonded and absolutely segregated CoN_4-NiN_4 sites optimized by DFT calculations (the orange, green, blue and gray spheres represent Ni, Co, N and C atoms, respectively).

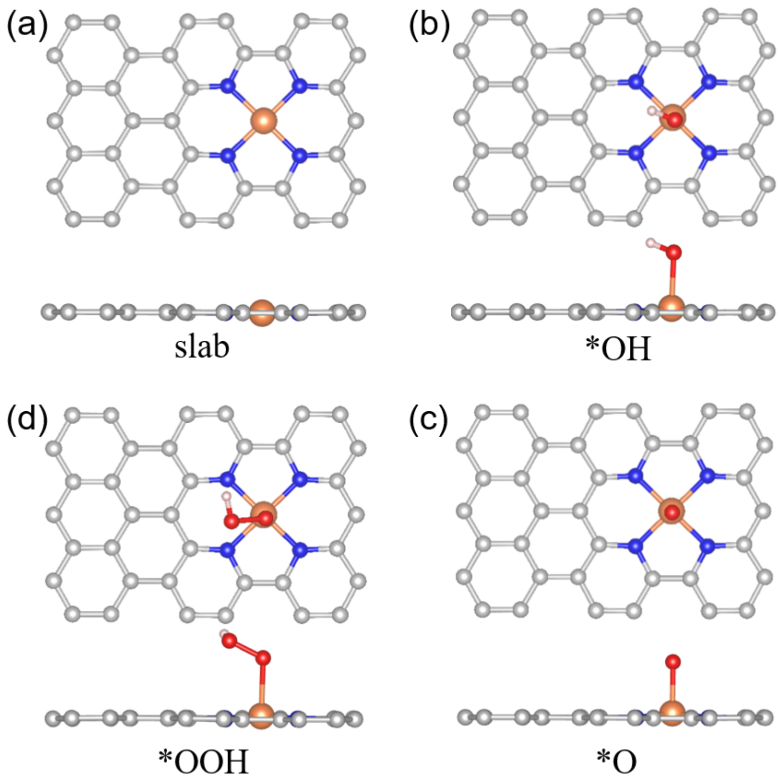

Figure S16. The structures of Ni-NC catalyst and three main adsorption intermediates on Ni-NC (orange: Ni, blue: N; red: O, white: H, gray: C).

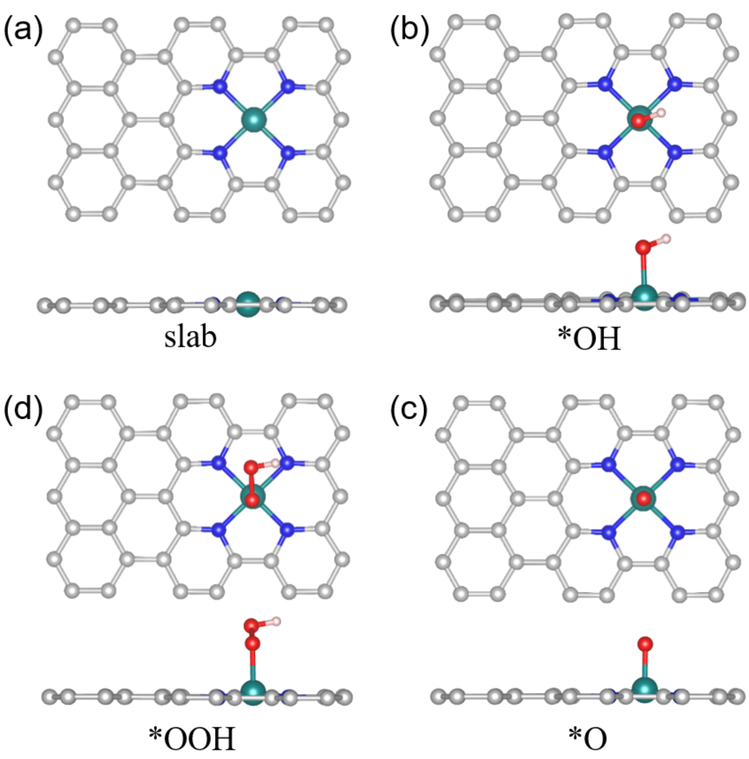

Figure S17. The calculated geometries and adsorption energies (in eV) of three main adsorption intermediates on Co-NC (green: Co, blue: N; red: O, white: H, gray: C).

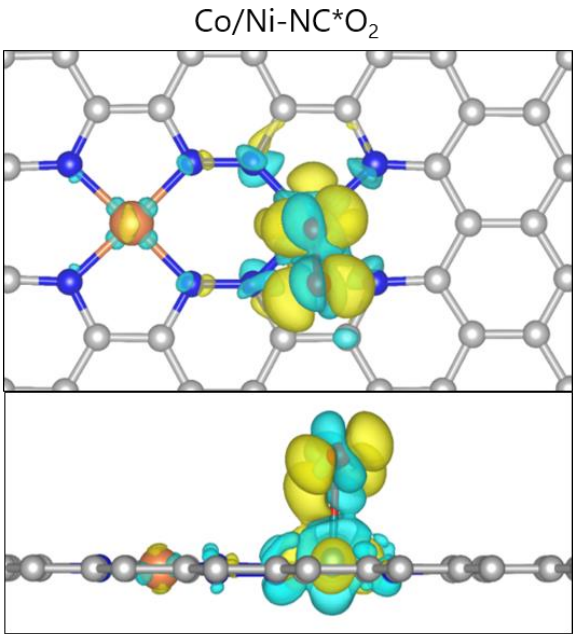

Figure S18. The calculated charge density difference of O_2 adsorbed on Co/Ni-NC with yellow and blue color referring to an increase and decrease of the electron density, respectively (iso-surface = 0.008 a.u.).

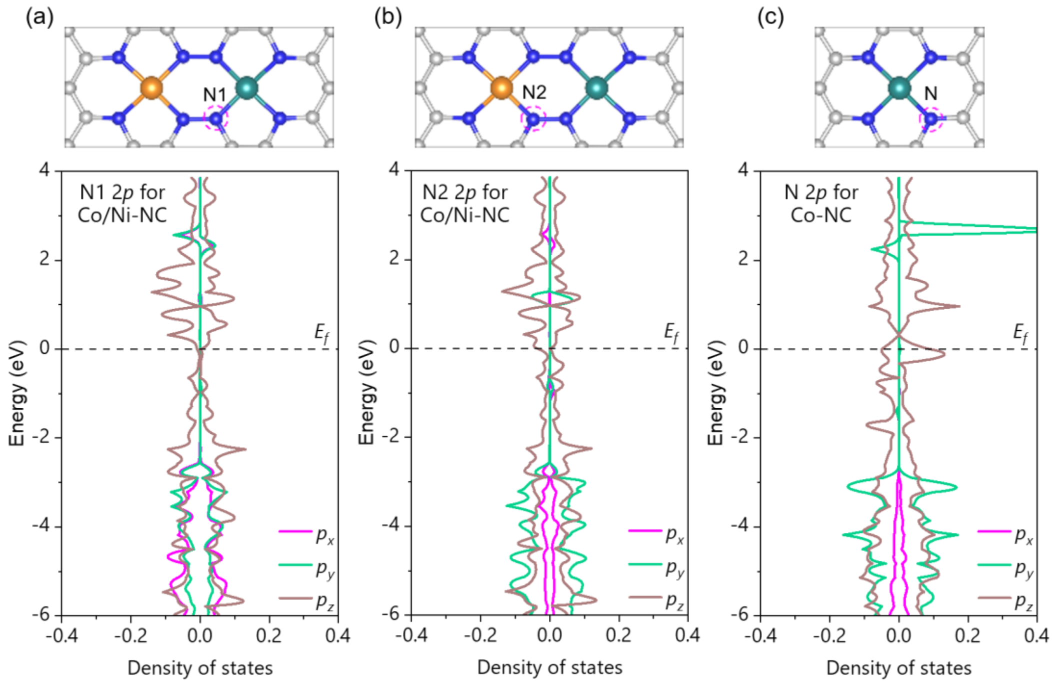

Figure S19. The calculated partial density of states (PDOS) of (a) N1 2*p* for Co/Ni-NC, (b) N2 2p for Co/Ni-NC and (c) N 2*p* for Co-NC.

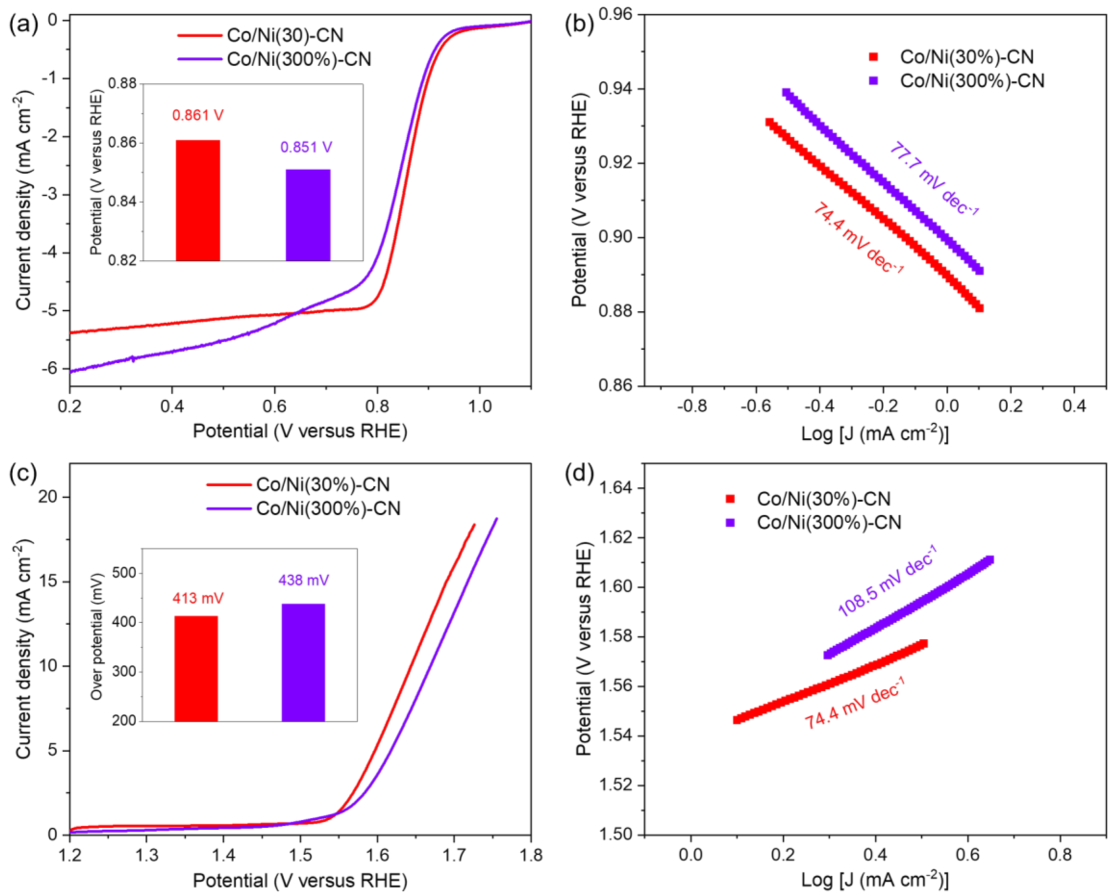

Figure S20. (a) ORR LSV curves collected under 1600 rpm in O_2 saturated 0.1 M KOH electrolyte (the insert is the half-wave potential $E_{1/2}$). (b) The corresponding Tafel plots obtained by the ORR LSV curves. (c) OER polarization curves obtained in 0.1 M KOH electrolyte (the insert is the overpotential: $E_{j=10}$ - 1.23 V). (d) Tafel plots obtained by the OER polarization curves.

***Ni/Co(x)-NC** (x = 300% or 30%, stands for the relative amount of the Ni^{2+}/Co^{2+} reactants used for the catalyst preparation, as described in the above Experimental Section) was also prepared for comparison. The Co/Ni loadings in Ni/Co(300%)-NC and Ni/Co(30%)-NC were determined to be 4.22/3.53 wt% and 0.68/0.56 wt% (by ICP-MS), respectively. Relative to the intermediate Co/Ni loadings (1.72/1.39 wt%, ICP-MS) of Ni/Co-NC, Ni/Co(300%)-NC and Ni/Co(30%)-NC may roughly represent Ni/Co(bonded)-NC and Ni/Co(segregated)-NC, respectively.

Table S1. EXAFS fitting parameters at the M *K*-edge for various samples.

Sample	Shell	CN^a	$R(Å)^b$	$\sigma^2(Å^2)^c$	$\Delta E_0(eV)^d$	*R* factor
Co *K*-edge (S_0^2=0.885)						

Co foil	Co-Co	12*	2.493±0.001	0.0063±0.0001	6.9±0.3	0.0009
CoO	Co-O	5.8±0.2	2.126±0.018	0.0091±0.0023	-3.0±2.7	0.0084
	Co-Co	12.1±1.1	3.008±0.005	0.0084±0.0006		
CoPc	Co-N	3.7±0.4	1.917±0.006	0.0025±0.0008	7.8±2.3	0.0062
	Co-C	6.9±0.9	2.934±0.015	0.0036±0.0020		
Co/Ni-NC	Co-N	4.3±0.7	1.897±0.011	0.0095±0.0016	-4.3±2.5	0.0129
	Co-Co	0.6±0.1	3.236±0.026	0.0081±0.0068		
Ni *K*-edge (S_0^2=0.887)						
Ni foil	Ni-Ni	12*	2.483±0.001	0.0061±0.0001	7.2±0.3	0.0013
NiO	Ni-O	6.0±0.2	2.076±0.007	0.0055±0.0008	-6.9±0.8	0.0064
	Ni-Ni	12.0±0.5	2.950±0.004	0.0060±0.0004		
NiPc	Ni-N	4.0±0.1	1.899±0.005	0.0028±0.0006	6.3±1.5	0.0086
	Ni-C	6.0±0.3	2.932±0.013	0.0021±0.0009		
Co/Ni-NC	Ni-N	4.1±0.6	1.832±0.014	0.0112±0.0047	5.3±0.3	0.0094

[a]*CN*, coordination number; [b]*R*, the distance to the neighboring atom; [c]σ^2, the Mean Square Relative Displacement (MSRD); [d]ΔE_0, inner potential correction; *R* factor indicates the goodness of the fit. *S*0^2 was fixed to 0.885 and 0.887, according to the experimental EXAFS fit of Co foil and Ni foil by fixing *CN* as the known crystallographic value.

Table S2. A comparison of the Zn-air battery performance of this work with the recent literatures.[8]

Catalysts	Open circuit voltage (V)	Peak power density (mW cm^{-2})	Specific capacity (mAh g_{Zn}^{-1}) @mA cm^{-1}	Cycling durability (h) @mA cm^{-2}	References
This work	1.55	155.9	771@10	340@2	

FeCo-DACs/NC	1.5	175		240@10	Adv. Mater. 2022, 34, 2107421
Fe/SNCFs-NH_3	1.38	255.8		1000@1	Adv. Mater. 2022, 34, 2105410
FeNi SAs/NC	1.45	42.2	779.4@5	45@1	Adv. Energy Mater. 2021, 11, 2101242
Pt-SCFP/C-12	1.40	122		80@5	Adv. Energy Mater. 2020, 10, 1903271
Co1-N3PS/HC	1.47	176	786@10	50	Angew. Chem., Int. Ed. 2021, 60, 3212
Fe-NP-Cl-C	1.495	260	812@10	175@10	Adv. Sci. 2024, 11, 2306599
Co-PorBpy-Co/CNT	1.482	159.4		~3@10	Adv. Sci. 2023, 10, 2206165
FeCoNC/SL	1.438	224.8	803@7.8	180@10	Adv. Sci. 2023, 10, 2205889
3DOM Fe/Co@NC-WO_{2-x}	1.46	165.1	757@10		Adv. Sci. 2022, 9, 2104237
FeNiCo|$MnGaO_x$/CNT	1.43	136.1	808.3	158@5	Small, 2023, 2308756
Fe-N/G-Co	1.54	98.8		112.5@10	Small Methods 2022, 6, 2101511
LaPNC	1.45	202	791.3@10	120@10	Energy Storage Mater. 2023, 54, 313
CoP_3/CeO_2/C-2	1.40	150	767.7@5	120@5	Appl. Catal. B-Environ. 2023, 321, 122029

Co/N/C-2.86 wt%	1.542	236	744@10	200@10	Chem. Eng. Sci. 2023, 273, 118654
FeMn-N/S-C-1000	1.45	346		90@10	J. Energy Chem. 2024, 90, 610
Mn_1@Fe-N-C/CNTs	1.53	249.3	778@50	200@10	ACS Nano. 2024, 18, 750
CoFe−N−C	1.49	142.1		200@5	Nano Lett. 2022, 22, 3392
FeCo/Se-CNT	1.543	173.4	745	70@5	Nano Lett. 2021, 21, 2255
Fe_1Co_3−NC-1100	1.479	372		190@10	ACS Catal. 2022, 12, 1216
IrCo-N-C	1.46	138.8		225@5	ACS Catal. 2021, 11, 8837
$Co_{0.25}Ni_{0.75}$@NCNT30	1.53	167		36@5	ACS Appl. Mater. Interfaces, 2021, 13, 30486
Fe_2-N_6-C (+RuO_2)	1.48	258.6	807.2@10	220@10	Inorg. Chem. 2023, 62, 5253
HM-Co@NC	1.48	209.4	770.2@20	120@2	J. Mater. Chem. A, 2022, 10, 10408
Co_1-PNC/Ni_1-PNC	1.14	252	874	45@10	Nano Res. 2021, 14, 3482

Table S3. The calculated adsorption energies of the intermediates at different sites in Co/Ni-NC, Co-NC, Ni-NC, Co/Ni(bonded)-NC and Co/Ni(segregated)-NC.

Configuration	intermediates	Adsorption energy (eV)
Co/Ni-NC (Ni site)	*OOH	-0.76
	*O	-2.54
	*OH	-1.79
Co/Ni-NC (Co site)	*OOH	-1.39
	*O	-3.67
	*OH	-2.45
Co-NC	*OOH	-1.51
	*O	-3.33
	*OH	-2.53
Ni-NC	*OOH	-0.51
	*O	-1.74
	*OH	-1.41
Co/Ni(bonded)-NC	*OOH	-1.80
	*O	-5.08
	*OH	-3.09
Co/Ni(segregated)-NC	*OOH	-1.43
	*O	-3.30
	*OH	-2.50

Table S4. The calculated Bader charges of metals in catalysts.

Configurations	Metal Sites	Metal Bader Charges |e|			
		*(Slab)	*OOH	*O	*OH

Co/Ni-NC	Co	+0.82	+1.03	+1.10	+1.06
	Ni	+0.84	+0.84	+0.83	+0.85
Co/Ni(bonded)-NC	Co	+0.65	+0.89	+0.96	+0.91
	Ni	+0.56	+0.82	+0.90	+0.81
Co/Ni(segregated)-NC	Co	+0.82	+1.05	+1.19	+1.07
	Ni	+0.83	+0.83	+0.83	+0.83
Co-NC	Co	+0.85	+1.04	+1.18	+1.06
Ni-NC	Ni	+0.84	+0.97	+1.08	+1.03

References

[1] a) B. Ravel, M. Newville, *J. Synchrotron Radiat.* **2005**, *12*, 537; b) S. Zabinsky, J. Rehr, A. Ankudinov, R. Albers, M. Eller, *Phys. Rev. B* **1995**, *52*, 2995.

[2] H. Funke, A. Scheinost, M. Chukalina, *Phys. Rev. B* **2005**, *71*, 094110.

[3] a) G. Kresse, J. Hafner, *Phys. Rev. B* **1993**, *47*, 558; b) G. Kresse, D. Joubert, *Phys. Rev. B* **1999**, *59*, 1758.

[4] J. P. Perdew, K. Burke, M. Ernzerhof, *Phys. Rev. Lett.* **1996**, *77*, 3865.

[5] a) S. Grimme, J. Antony, S. Ehrlich, H. Krieg, *J. Chem. Phys.* **2010**, *132*, 154104; b) A. Tkatchenko, M. Scheffler, *Phys. Rev. Lett.* **2009**, *102*, 073005.

[6] H. J. Monkhorst, J. D. Pack, *Phys. Rev. B* **1976**, *13*, 5188.

[7] J. K. Nørskov, J. Rossmeisl, A. Logadottir, L. Lindqvist, J. R. Kitchin, T. Bligaard, H. Jonsson, *J. Phys. Chem. B* **2004**, *108*, 17886.

[8] a) Y. He, X. Yang, Y. Li, L. Liu, S. Guo, C. Shu, F. Liu, Y. Liu, Q. Tan, G. Wu, *Acs Catal.* **2022**, *12*, 1216; b) J. Ji, L. Wu, S. Zhou, T. Qiu, Z. Li, L. Wang, L. Zhang, L. Ma, M. Ling, S. Zhou, *Small Methods* **2022**, *6*, 2101511; c) B. Hu, A. Huang, X. Zhang, Z. Chen, R. Tu, W. Zhu, Z. Zhuang, C. Chen, Q. Peng, Y. Li, *Nano Res.* **2021**, *14*, 3482; d) X.

Zhou, J. Gao, Y. Hu, Z. Jin, K. Hu, K. M. Reddy, Q. Yuan, X. Lin, H.-J. Qiu, *Nano Lett.* **2022**, *22*, 3392; e) L. Yang, X. Zhang, L. Yu, J. Hou, Z. Zhou, R. Lv, *Adv. Mater.* **2022**, *34*, 2105410; f) D. Yu, Y. Ma, F. Hu, C.-C. Lin, L. Li, H.-Y. Chen, X. Han, S. Peng, *Adv. Energy Mater.* **2021**, *11*, 2101242; g) X. Wang, J. Sunarso, Q. Lu, Z. Zhou, J. Dai, D. Guan, W. Zhou, Z. Shao, *Adv. Energy Mater.* **2020**, *10*, 1903271; h) Y. Chen, R. Gao, S. Ji, H. Li, K. Tang, P. Jiang, H. Hu, Z. Zhang, H. Hao, Q. Qu, *Angew. Chem. Int. Ed.* **2021**, *60*, 3212; i) L. Luo, Y. Liu, S. Chen, Q. Zhu, D. Zhang, Y. Fu, J. Li, J. Han, S. Gong, *Small* **2023**, 2308756; j) Z. Bi, H. Zhang, X. Zhao, Y. Wang, F. Tan, S. Chen, L. Feng, Y. Zhou, X. Ma, Z. Su, X. Wang, T. Wågberg, G. Hu, *Energy Storage Mater.* **2023**, *54*, 313; k) J. Li, Y. Kang, Z. Lei, P. Liu, *Appl. Catal. B-Environ.* **2023**, *321*, 122029; l) L. Luo, Y. Xu, D. Wang, X. Qiu, *Chem. Eng. Sci.* **2023**, *273*, 118654; m) Y. He, H. Li, Y. Wang, Y. Jia, Y. Liu, Q. Tan, *J. Energy Chem.* **2024**, *90*, 610; n) L. Ran, Y. Xu, X. Zhu, S. Chen, X. Qiu, *ACS nano* **2023**, *18*, 750; o) M. Xiao, J. Zhu, S. Li, G. Li, W. Liu, Y.-P. Deng, Z. Bai, L. Ma, M. Feng, T. Wu, *ACS Catal.* **2021**, *11*, 8837; p) H. Zhang, M. Zhao, H. Liu, S. Shi, Z. Wang, B. Zhang, L. Song, J. Shang, Y. Yang, C. Ma, *Nano Lett.* **2021**, *21*, 2255; q) A. Kundu, A. Samanta, C. R. Raj, *ACS Appl. Mater. Interfaces* **2021**, *13*, 30486; r) Y. Xu, W. Li, L. Chen, W. Li, W. Feng, X. Qiu, *Inorg. Chem.* **2023**, *62*, 5253; s) M. Huo, T. Sun, Y. Wang, P. Sun, J. Dang, B. Wang, N. A. Dharanipragada, A. K. Inge, W. Zhang, R. Cao, *J. Mater. Chem. A* **2022**, *10*, 10408; t) X. Tan, J. Zhang, F. Cao, Y. Liu, H. Yang, Q. Zhou, X. Li, R. Wang, Z. Li, H. Hu, Q. Zhao, M. Wu, *Adv. Sci.* **2024**, *11*, 2306599; u) X. Zhao, J. Chen, Z. Bi, S. Chen, L. Feng, X. Zhou, H. Zhang, Y. Zhou, T. Wågberg, G. Hu, *Adv. Sci.* **2023**, *10*, 2205889; v) J. Li, P. Liu, J. Yan, H. Huang, W. Song, *Adv. Sci.* **2023**, *10*, 2206165; w) Q. Han, X. Zhao, Y. Luo, L. Wu, S. Sun, J. Li, Y. Wang, G. Liu, Z. Chen, *Adv. Sci.* **2022**, *9*, 2104237.
